# Endophilin-A2 dependent VEGFR2 endocytosis promotes sprouting angiogenesis

**DOI:** 10.1038/s41467-019-10359-x

**Published:** 2019-05-28

**Authors:** Gael Genet, Kevin Boyé, Thomas Mathivet, Roxana Ola, Feng Zhang, Alexandre Dubrac, Jinyu Li, Nafiisha Genet, Luiz Henrique Geraldo, Lorena Benedetti, Steffen Künzel, Laurence Pibouin-Fragner, Jean-Leon Thomas, Anne Eichmann

**Affiliations:** 1 Cardiovascular Research Center, Department of Internal Medicine, Yale University School of Medicine, New Haven, CT 06511 USA; 2 Inserm U970, Paris Cardiovascular Research Center, Paris, 75015 France; 3 Department of Neuroscience and Cell Biology, School of Medicine, Yale University School of Medicine, New Haven, CT 06511 USA; 4 Department of Neurology, Yale University School of Medicine, New Haven, CT 06511 USA; 5 Sorbonne Universités, UPMC Université Paris 06, Institut National de la Santé et de la Recherche Médicale U1127, Centre National de la Recherche Scientifique, AP-HP, Institut du Cerveau et de la Moelle Epinière, Hôpital Pitié-Salpêtrière, Paris, France; 6 Department of Cellular and Molecular Physiology, Yale University School of Medicine, New Haven, CT 06511 USA; 7 Present Address: Functional Genomics, Proteomics and Experimental Pathology Department, Prof. Dr. I. Chiricuta Oncology Institute, Cluj-Napoca, Romania, Department of Basic, Preventive and Clinical Science, University of Transylvania, Brasov, Romania

**Keywords:** Growth factor signalling, Endocytosis, Angiogenesis

## Abstract

Endothelial cell migration, proliferation and survival are triggered by VEGF-A activation of VEGFR2. However, how these cell behaviors are regulated individually is still unknown. Here we identify Endophilin-A2 (ENDOA2), a BAR-domain protein that orchestrates CLATHRIN-independent internalization, as a critical mediator of endothelial cell migration and sprouting angiogenesis. We show that *EndoA2* knockout mice exhibit postnatal angiogenesis defects and impaired front-rear polarization of sprouting tip cells. ENDOA2 deficiency reduces VEGFR2 internalization and inhibits downstream activation of the signaling effector PAK but not ERK, thereby affecting front-rear polarity and migration but not proliferation or survival. Mechanistically, VEGFR2 is directed towards ENDOA2-mediated endocytosis by the SLIT2-ROBO pathway via SLIT-ROBO-GAP1 bridging of ENDOA2 and ROBO1. Blocking ENDOA2-mediated endothelial cell migration attenuates pathological angiogenesis in oxygen-induced retinopathy models. This work identifies a specific endocytic pathway controlling a subset of VEGFR2 mediated responses that could be targeted to prevent excessive sprouting angiogenesis in pathological conditions.

## Abstract (web summary)

VEGF-A/VEGFR2 signaling is a key driver of endothelial cell migration during sprouting angiogenesis. Here Genet et al. show that endophilin A2 regulates these processes by mediating clathrin-independent VEGFR2 internalization.

## Introduction

Vascular endothelial growth factor A (VEGF) is a secreted polypeptide that is critical for vascular development, angiogenesis and arteriogenesis^1–4^. VEGF exerts its action by binding to the receptor tyrosine kinase VEGF receptor 2 (VEGFR2; also known as KDR or FLK1)^5^, which is expressed mainly in endothelial cells (ECs), but also in some neuronal cell populations^6^. VEGF binding to VEGFR2 triggers receptor dimerization and phosphorylation of tyrosine residues in the cytoplasmic kinase domain, in turn activating various intracellular cascades, including PI3K/AKT, MAPK, SRC, and PAK signaling, to mediate survival, proliferation, and migration^7–9^. A major challenge in the field is to determine how these cascades instruct specific EC behaviors.

Endocytosis and subsequent receptor signaling from endosomal compartments have emerged as major determinants of signaling output^10,11^. Endosomes are distributed to various intracellular locations via microtubules. The sorting and trafficking processes of these small vesicles provide time for protein–protein interactions and assembly of signaling complexes^12–14^. Thus, the knowledge of events involved in receptor endocytosis and trafficking is essential to understand the regulation of its activity.

Receptor signaling is initiated by endocytic uptake into the cell. Until now, the major known endocytic route for VEGFR2 was thought to be via the CLATHRIN-mediated endocytic (CME) pathway^10,15,16^. CLATHRIN-mediated VEGFR2 endocytosis is facilitated by various extracellular cues, including the guidance receptors EPHRIN-B2 and NEUROPILIN-1 (NRP1), which both promote CME uptake and intracellular VEGFR2 trafficking^17–22^. However, several studies reported that VEGF-induced internalization or signaling persisted upon inhibition of CME^23–26^, suggesting that the receptor might also be internalized through CLATHRIN-independent endocytic routes.

Fast endophilin-mediated endocytosis (FEME) is a newly discovered CLATHRIN-independent endocytosis pathway^27–31^. ENDOPHILIN proteins are cytoplasmic Bin/Amphiphysin/Rvs (BAR)-domain-containing proteins involved in the formation and scission of endocytic vesicles^32–34^. FEME mediates internalization of Shiga and Cholera toxins, which are well known to enter cells independently of CME^29^. FEME is triggered upon activation of specific receptors by their cognate ligands^29^. Receptor activation leads to the conversion of phospholipid molecules PI(4,5)P_2_ to PI(3,4)P_2_, which induces the recruitment of ENDOPHILIN to the plasma membrane and the formation of endocytic vesicles named ENDOPHILIN positive assemblies (EPA)^27^. FEME occurs preferentially at the lamellipodia of migrating cells and mediates ligand-dependent uptake of several G protein-coupled receptors and receptor tyrosine kinases in vitro^27^. Whether FEME contributes to vascular development in vivo remained unknown.

Here we show that ENDOA2 selectively regulates EC migration during postnatal and pathological angiogenesis by controlling CME-independent VEGFR2 endocytosis and activation of downstream PAK, but not ERK signaling. Targeting of VEGFR2 toward ENDOA2 is controlled by the SLIT-ROBO guidance pathway, which has been previously identified as a regulator of polarized endothelial migration^9,35^. Altogether, this work reveals an endocytic mechanism regulating sprouting angiogenesis, opening avenues to selectively target this cell behavior.

## Results

### ENDOA2 regulates angiogenesis in the postnatal mouse retina

**Fig. 1:**
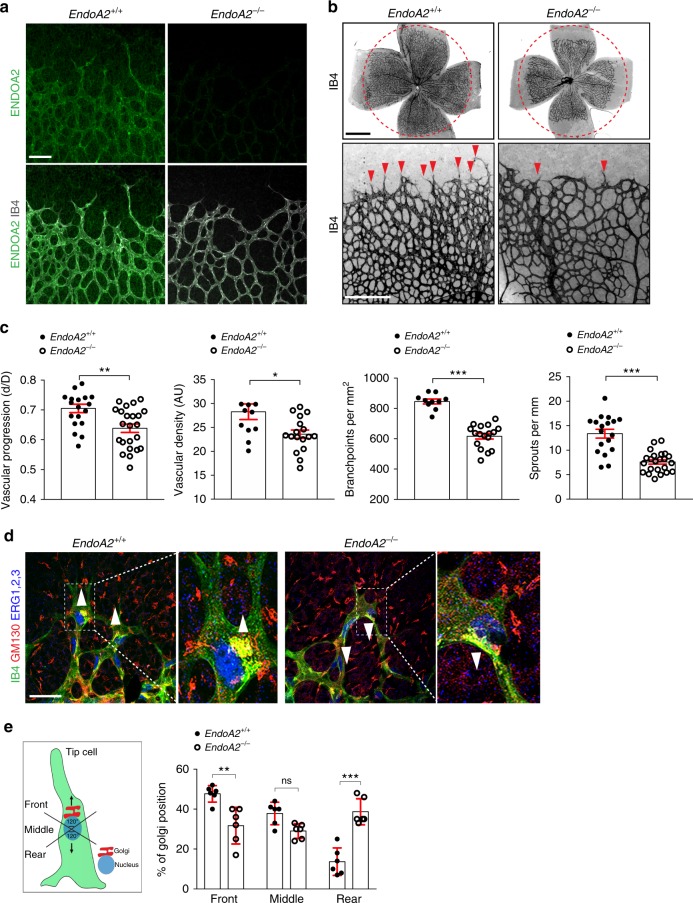
ENDOA2 regulates postnatal mouse retina angiogenesis. **a** ENDOA2 and IB4 staining in retinal flatmounts from P5 mice. Note enrichment of ENDOA2 in IB4 + wild-type vessels, and absence of ENDOA2 expression in *EndoA2*^−/−^ mice. **b** IB4 stained P5 retinal flatmounts of *EndoA2*^*+/+*^ and *EndoA2*^−/−^ mice. The dashed circles outline vascular coverage in wild-type retina. Red arrowheads show tip cell sprouting. **c** Quantification of vascular progression (d: vascular coverage length; D: retinal petal length), vessel density, number of branchpoints and vessel sprouts (*N* = 10–23 retinas/group, *t*-test: *P < 0.05, **P < 0.01, ***P < 0.001). **d** IB4, GM130, and ERG1/2/3 labeling shows Golgi orientation (white arrowheads) of P5 tip cells in *EndoA2*^*+/+*^ and *EndoA2*^−/−^ retinas. Boxed areas are magnified in the right panels. **e** Schematic of Golgi positions in tip cells and quantification of the Golgi position in retinas shown in **d** (*N* = 6 retinas per group, at least 50 tip cells per retina were quantified; two-way ANOVA: ns *P* > 0.05, **P < 0.01, ***P < 0.001). Error bars represent mean ± s.e.m. Scale bars: **a** 100 μm, **b** (upper panel) 1 mm, **b** (lower panel) 300 μm, **d** 50 μm

Gene expression analysis of *ENDOA1, 2*, and *3* isoforms (*SH3GL2*, *SH3GL1*, and *SH3GL3*, respectively) in purified ECs from various sources showed that *ENDOA2* was the only isoform expressed in this cell type (Supplementary Fig. 1a–c). Immunostaining of retinas from P5 wild-type mice showed that ENDOA2 labeled the endothelium, with lower expression in the neuronal retinal layers and in perivascular mural cells and astrocytes (Fig. 1a, Supplementary Fig. 1d–f). Furthermore, measurement of mRNA levels in retinal non-ECs revealed that in addition to low levels of *EndoA2*, these cells also expressed equal levels of *EndoA1* and *EndoA3* (Supplementary Fig. 1g). These data suggested a unique role of ENDOA2 in ECs, while it may function redundantly with ENDOA1 and A3 in non-ECs, as previously shown in neurons^36,37^.

To test the function of ENDOA2 in vascular development, we used *Sh3gl1*^−/−^ mice (hereafter named *EndoA2*^−/−^)^36^, which were viable and recovered at expected Mendelian ratios at birth (Supplementary Fig. 2a). *EndoA2*^−/−^ mice did not show any detectable *EndoA2* expression in retinas or mouse lung endothelial cells (mLECs) (Fig. 1a, Supplementary Fig. 2b), and *EndoA2* gene deletion did not affect *EndoA1* or *EndoA3* gene expression (Supplementary Fig. 2c, d).

We analyzed the embryonic hindbrain and the postnatal mouse retina vasculature, two convenient models that allow detecting even subtle effects on angiogenesis^38^. Vessel morphology at embryonic day 11.5 was similar between wild-type and *EndoA2*^−/−^ littermates (Supplementary Fig. 2e–g). In contrast, *EndoA2* deletion significantly reduced vascular radial expansion, vessel density and branching in the postnatal retina (Fig. 1b, c). *EndoA2* deletion did not affect the neuronal layers beneath the retinal vasculature, nor astrocyte, pericyte or smooth muscle coverage (Supplementary Fig. 3). Production and localization of growth factors such as VEGF and SLIT2 were similar between *EndoA2*^−/−^ mice and wild-type littermates (Supplementary Fig. 4). Overall, these data support a selective function for ENDOA2 in postnatal ECs.

*EndoA2*^−/−^ retinas displayed lower overall numbers of ERG1,2,3 positive ECs but similar endothelial EdU incorporation and cell size (Supplementary Fig. 5). Vessel stability was not affected, as shown by similar area of collagen IV-positive, IB4-negative empty basement membrane sleeves between wild-type and *EndoA2*^−/−^ littermates (Supplementary Fig. 6a). However, the numbers of angiogenic sprouts at the vascular front of *EndoA2*^−/−^ mutant mice were severely decreased in comparison to wild-type littermates (Fig. 1b). Acquisition of tip cell front-rear polarity is a key regulator of sprouting; therefore, we analyzed front-rear polarity by staining P5 retinas with IB4, ERG1,2,3 and an anti-GM130 antibody to label the Golgi apparatus. In wild-type littermates, around 45% of tip cells had their Golgi positioned toward the leading edge, while around 40% of *EndoA2*^−/−^ tip cells had their Golgi positioned behind the nucleus away from the migration front (Fig. 1d, e), demonstrating impaired front-rear polarization in *EndoA2*^−/−^ tip cells. Apico-basal polarity of ECs in the stalk position appeared normal as shown by podocalyxin staining of the luminal membrane (Supplementary Fig. 6b). Sprouting defects in *EndoA2*^−/−^ mice persisted until P12 and impaired formation of the deeper retinal vasculature layer (Supplementary Fig. 6c). Together, these data show that ENDOA2 is required for postnatal sprouting angiogenesis by regulating tip cell polarization and endothelial migration, although we cannot exclude that reduced cell cycling may contribute to the lower EC numbers in ENDOA2 deficient retinas.

### ENDOA2 controls CLATHRIN-independent VEGFR2 internalization

**Fig. 2:**
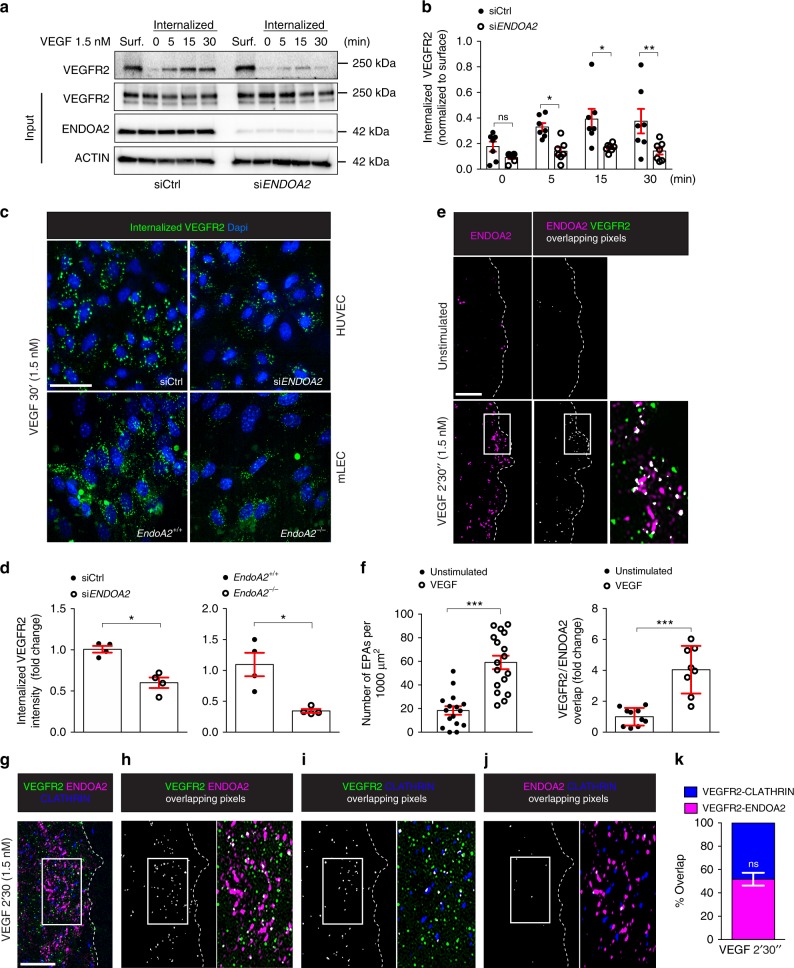
ENDOA2 mediates CLATHRIN-independent VEGFR2 internalization. **a** Cell surface biotinylation assay of VEGFR2 internalization in response to VEGF in Control siRNA (siCtrl) and *ENDOA2* siRNA silenced HUVECs. VEGFR2, ENDOA2, and ACTIN expression in the total cell lysate are shown (input). Surf: surface expression of VEGFR2 before ligand stimulation and stripping. **b** Quantification of internalized VEGFR2 normalized to VEGFR2 surface expression (*N* = 7 independent experiments; two-way ANOVA: ns *P* > 0.05, *P < 0.05, **P < 00.1). **c** Antibody feeding assay of VEGFR2 internalization in response to VEGF (1.5 nM, 30 min) in Ctrl and *ENDOA2* siRNA silenced HUVECs or mLECs isolated from *EndoA2*^*+/+*^ and *EndoA2*^−/−^ mice. **d** Quantification of internalized VEGFR2 fluorescence in **c** (*N* = 4 independent experiments, at least 10^3^ cells analyzed per experiment; Mann–Whitney U test: *P < 0.05). **e** SIM images of HUVEC lamellipodia stained for ENDOA2 and VEGFR2 before and after VEGF stimulation (1.5 nM for 2′30″). **f** Left panel shows quantification of EPA number at the lamellipodia (*N* = 16–17 cells/group analyzed from three independent experiments; *t*-test and Mann–Whitney U test: ***P < 0.001). Right panel shows quantification of pixel overlap between VEGFR2 and ENDOA2 fluorescent signals (*N* = 8–10 cells/group analyzed from three independent experiments; Mann–Whitney U test: ***P < 0.001). **g** SIM image of HUVEC lamellipodia stained for ENDOA2, VEGFR2 and CLATHRIN heavy chain after VEGF stimulation (1.5 nM for 2′30″). **h** Overlapping pixels between VEGFR2/ENDOA2, **i** overlapping pixels between VEGFR2/CLATHRIN, and **j** overlapping pixels between ENDOA2/CLATHRIN from the image presented in **g** are shown in white. Boxed areas are magnified to highlight VEGFR2/ENDOA2, VEGFR2/CLATHRIN, or ENDOA2/CLATHRIN overlaps (white). **k** Quantification of overlap between VEGFR2/ENDOA2 and VEGFR2 /CLATHRIN fluorescent signals (*N* = 8–10 cells per group analyzed from three independent experiments; Mann–Whitney U test: ns *P* > 0.05). Error bars represent mean ± s.e.m. Scale bars: **c** 20 μm, **e** 2 μm, **g** 1 μm

Since tip cell migration is controlled by VEGFR2^3–7^, and ENDOA2 has been implicated in VEGF endocytosis^27^, we determined whether ENDOA2 loss of function affected VEGFR2 internalization. HUVECs express ENDOA2 around the nucleus, at the plasma membrane and in intracellular punctae called EPAs carrying internalized receptors^27^, and *ENDOA2* siRNA silencing abolished ENDOA2 expression (Supplementary Fig. 7a, b). Cell surface biotinylation assay showed that *ENDOA2* siRNA decreased VEGFR2 internalization induced by VEGF by about 50% (Fig. 2a, b). Next, we used an antibody feeding assay where HUVECs or mLECs were incubated with an antibody binding to the extracellular domain of VEGFR2 prior to VEGF stimulation, then stripped, fixed, and labeled with a secondary antibody. Again, *ENDOA2* knockdown decreased VEGFR2 internalization in both EC types (Fig. 2c, d). No VEGFR2 internalization was detected after PBS treatment and antibody specificity was validated using *VEGFR2 (KDR)* siRNA in HUVECs (Supplementary Fig. 7c, d). To further characterize VEGFR2 endocytosis and trafficking via ENDOA2, we used super-resolution structured illumination microscopy (SIM)^39^, which allows quantification of the proximity between two proteins (0–200 nm) (Supplementary Fig. 8). SIM imaging revealed that VEGF stimulation induced formation of EPAs underneath the plasma membrane of HUVEC lamellipodia (Fig. 2e, f). VEGF stimulation promoted overlap between VEGFR2 and ENDOA2 at the leading edge of the cell (Fig. 2e, f), indicating that VEGF targeted VEGFR2 into ENDOA2 positive vesicles. SIM analysis showed that after VEGF stimulation, VEGFR2 overlapped with either CLATHRIN HEAVY CHAIN (CHC) (48.02 ± 7%, *n* = 8 different cells) or ENDOA2 (51.98 ± 7%, *n* = 8 different cells) (Fig. 2g–k). The overlap between VEGFR2 and both ENDOA2 and CHC fluorescent signals within a same complex was negligible (2.9±0.89%, *n* = 8 different cells) (Fig. 2j, k), indicating that VEGF induces VEGFR2 internalization through distinct CLATHRIN or ENDOA2-mediated endocytosis pathways. In support of this idea, *ENDOA2* silencing did not affect CLATHRIN-mediated VEGFR2 internalization following VEGF stimulation (Supplementary Fig. 9). These data suggest that ENDOA2 mediates CLATHRIN-independent VEGFR2 endocytosis in ECs.

### ENDOA2 promotes VEGF-induced endothelial migration

**Fig. 3:**
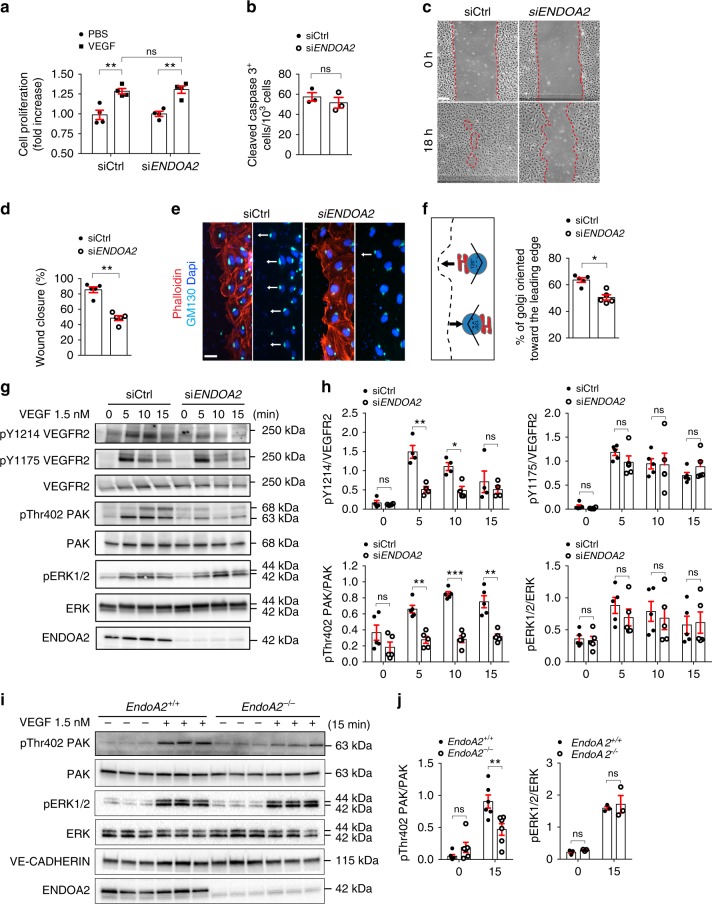
ENDOA2 controls endothelial cell migration and polarity. **a** VEGF-induced cell proliferation (6 nM, 48 h stimulation) assessed by XCelligence system in siControl (Ctrl) and si*ENDOA2* silenced HUVECs (*N* = 4 independent experiments; two-way ANOVA: ns *P* > 0.05, **P < 0.01). **b** Cleaved caspase-3 staining of HUVECs cultured in 0.5% FBS for 24 h (*N* = 3 independent experiments; Mann–Whitney U test: ns *P* > 0.05). **c** HUVEC scratch wound migration in response to VEGF (3 nM). **d** Quantification of wound closure shown in **c** (*N* = 5 independent experiments; Mann–Whitney U test: **P < 0.01). **e** Phalloidin, Dapi, and GM130 Golgi labeling at the scratch wound edge to assess Golgi polarization in front of the nucleus (arrows indicate direction of migration) in response to VEGF (3 nM, 2 h). **f** Quantification of Golgi orientation (*N* = 5 independent experiments; Mann–Whitney U test: *P < 0.05). **g** Western-blot analysis of phosphorylation of the indicated proteins in response to VEGF (1.5 nM) in HUVECs. **h** Quantification of phosphorylation normalized to total protein levels (*N* = 4–5 independent experiments; two-way ANOVA: *P < 0.05, **P < 0.01, ***P < 0.001, ns *P* > 0.05). **i** Western-blot analysis of VEGF-induced (1.5 nM) PAK and ERK phosphorylation in mLEC from *EndoA2*^*+/+*^ and *EndoA2*^−/−^ mice. Each lane represents one mouse. **j** Quantification of phosphorylation normalized to total proteins (*N* = 3–5 mice; two-way ANOVA: ns *P* > 0.05, **P < 0.01). Error bars represent mean ± s.e.m. Scale bars: **c** 200 μm, **e** 25 μm

VEGF signaling through VEGFR2 activates EC proliferation, survival, and migration, leading us to examine VEGF responses in *ENDOA2* knockdown HUVECs and *Endo*A2^−/−^ mLECs. As seen in vivo in *EndoA2* deficient retinas, *ENDOA2* silencing failed to affect VEGF-induced cell proliferation or cell death in vitro (Fig. 3a, b). However, *ENDOA2* silencing modified the morphology of HUVECs by increasing cellular area and promoting cell spreading (Supplementary Fig. 10a, b). *ENDOA2* silenced HUVECs exhibited more F-actin stress fibers and increased phospho-myosin light chain 2 staining (pMLC2) (Supplementary Fig. 10a, c) which are common features of cells harboring migration defects^40–42^. *ENDOA2* knockdown indeed inhibited VEGF-induced cell migration in a scratch wound assay (Fig. 3c, d) and impaired VEGF-induced Golgi polarization toward the leading edge (Fig. 3e, f). At the molecular level, *ENDOA2* siRNA inhibited phosphorylation of the VEGFR2 Y1214 site as well as PAK and p38 activation in response to VEGF, but did not affect VEGFR2 Y1175 phosphorylation and downstream ERK activation (Fig. 3g, h and Supplementary Fig. 10d, e). Similarly, reduced pPAK but not ERK activation in response to VEGF was seen in mLECs from *EndoA2*^−/−^ mice when compared with wild-type littermates (Fig. 3i, j). ERK activation in response to VEGF occurs downstream of CLATHRIN-mediated VEGFR2 endocytosis^15^, further supporting that ENDOA2 affects a subset of CLATHRIN-independent VEGF downstream signaling events leading to polarized EC migration.

### SLIT2/ROBO1 targets VEGFR2 to ENDOA2-dependent endocytosis

**Fig. 4:**
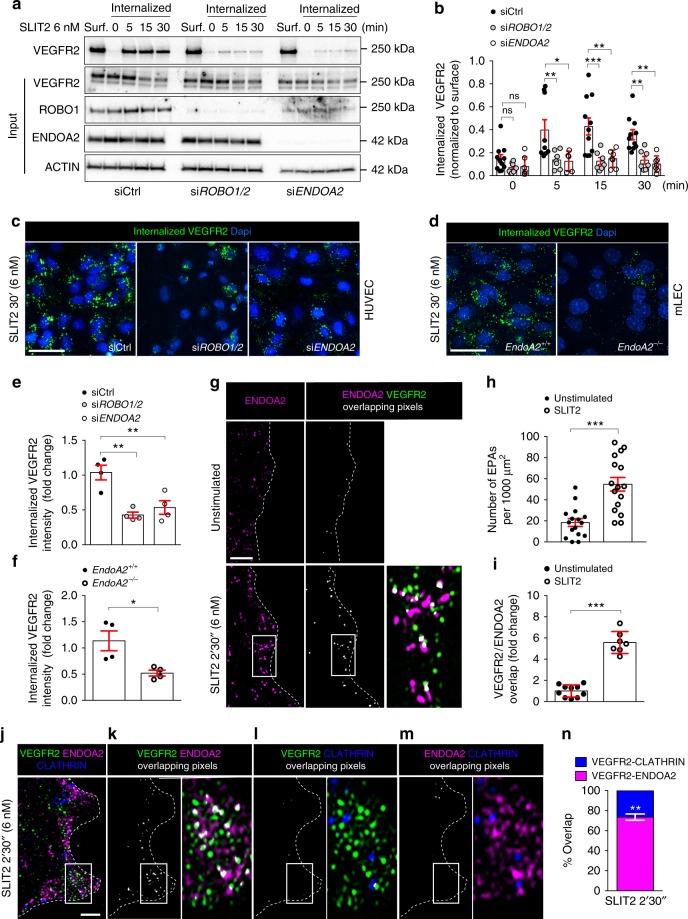
SLIT2 induces ENDOA2-dependent VEGFR2 internalization. **a** Cell surface biotinylation assay of VEGFR2 internalization in response to SLIT2 (6 nM) in Control (Ctrl), *ROBO1/2* and *ENDOA2* siRNA silenced HUVECs. VEGFR2, ROBO1, ENDOA2, and ACTIN expression from the total cell lysate are shown as loading controls (input). Surf: surface expression. **b** Quantification of internalized VEGFR2 normalized to VEGFR2 surface expression before stimulation (*N* = 7 independent experiments; two-way ANOVA test: *P < 0.05, **P < 0.01, ***P < 0.001, ns *P* > 0.05). **c**, **d** Antibody feeding assay to measure VEGFR2 internalization in response to SLIT2 (6 nM) in *Ctrl*, *ROBO1/2*, and *ENDOA2* siRNA silenced HUVECs (**c**) and mLECs from *EndoA2*^*+/+*^ and *EndoA2*^−/−^ mice (**d**). **e**, **f** Quantification of internalized VEGFR2 fluorescent intensity shown in **c** and **d**, respectively (*N* = 4 independent experiment, at least 10^3^ cells analyzed per experiment; **e** one-way ANOVA and **f** Mann–Whitney U test: *P < 0.05). **g** SIM images of HUVEC lamellipodia stained for ENDOA2 before and after SLIT2 stimulation (6 nM for 2′30″). **h** SLIT2 effects on EPA formation at the cell migration front (*N* = 16–17 cells per group analyzed from three independent experiments; *t*-test: ***P < 0.001). **i** SLIT2 increases the pixel overlap between VEGFR2 and ENDOA2 fluorescent signals (*N* = 8–10 cells per group analyzed from three independent experiments; Mann–Whitney U test: ***P < 0.001). **j** SIM images of HUVEC lamellipodia stained for ENDOA2, VEGFR2, and CLATHRIN heavy chain after SLIT2 stimulation (6 nM for 2′30″). **k**–**m** Overlapping pixels between VEGFR2/ENDOA2 (**k**), VEGFR2/CLATHRIN (**l**), and ENDOA2/CLATHRIN (**m**) from the image presented in **j** are shown in white. Boxed areas are magnified to highlight VEGFR2/ENDOA2, VEGFR2/CLATHRIN, or ENDOA2/CLATHRIN overlap (white). **n** Quantification of overlap between VEGFR2/ENDOA2 and VEGFR2/CLATHRIN fluorescent signals (*N* = 8–10 cells per group analyzed from three independent experiments; Mann–Whitney U test: **P < 0.01) (right panel). Error bars represent mean ± s.e.m. Scale bars: **c** and **d** 20μm, **g** 2μm, **j** 1μm

We next determined mechanisms directing VEGFR2 toward the ENDOA2-mediated endocytosis pathway. We had previously observed that treatment of HUVECs with the SLIT2 ligand induced VEGFR2 internalization in a *ROBO1* and *ROBO2* dependent manner^35^. To test if SLIT2 was involved in ENDOA2-mediated VEGFR2 endocytosis, we stimulated HUVECs with recombinant SLIT2 protein followed by cell surface biotinylation or antibody feeding assays to assess VEGFR2 internalization. In both assays, we found that SLIT2 promoted VEGFR2 internalization, and that both *ROBO1/ROBO2* and *ENDOA2* siRNAs inhibited this process (Fig. 4a–c, e). Likewise, mLECs from *EndoA2*^−/−^ mice exhibited impaired SLIT2-induced VEGFR2 internalization (Fig. 4d, f). SLIT2 stimulation promoted EPA formation at the lamellipodia (Fig. 4g, h) and stimulated overlap between VEGFR2 and ENDOA2 at the leading edge of the cell (Fig. 4g, i). Triple staining with antibodies against ENDOA2, VEGFR2 and CHC showed that SLIT2 promoted VEGFR2 endocytosis preferentially via the ENDOA2 pathway (74.25±4.9%) compared with CME (25.27±4.9%) (Fig. 4j–n). Thus, SLIT2-ROBO1/2 signaling promoted ENDOA2-mediated VEGFR2 endocytosis.

**Fig. 5:**
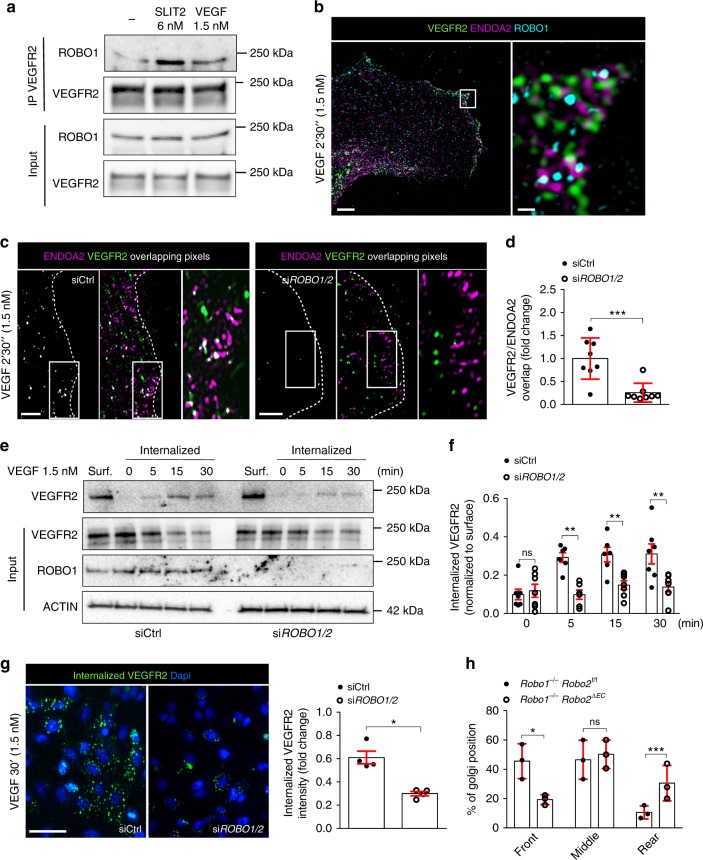
ROBO1 promotes VEGF-induced ENDOA2-mediated VEGFR2 endocytosis. **a** VEGFR2 immunoprecipitation in HUVEC after VEGF (1.5 nM for 2′30″) or SLIT2 (3 nM for 2′30″) stimulation, and western blot for ROBO1 and VEGFR2. ROBO1 and VEGFR2 expression from the total cell lysate are shown as loading controls (input). **b** SIM image of HUVECs stained for VEGFR2, ENDOA2, and ROBO1 after VEGF stimulation (1.5 nM for 2′30″). Right panel is a higher magnification of the boxed area in the left panel. **c** SIM images of the lamellipodia of Ctrl and *ROBO1/2* siRNA treated HUVEC stained for ENDOA2 and VEGFR2 after VEGF stimulation (1.5 nM for 2′30″). Overlapping pixels between VEGFR2/ENDOA2 fluorescent signals are shown in white. Boxed areas are magnified to highlight VEGFR2/ENDOA2 proximity. **d** Quantification of pixel overlap between VEGFR2 and ENDOA2 fluorescent signals (*N* = 8 cells per group analyzed from three independent experiments; Mann–Whitney U test: ***P < 0.01). **e** Western-blot analysis of VEGF-induced VEGFR2 internalization (3 nM) from cell surface biotinylation assay in *Ctrl* or *ROBO1/2* siRNA silenced HUVECs. VEGFR2, ROBO1, and ACTIN expression from the total cell lysate are shown as loading controls (input). Surf: surface expression. **f** Quantification of internalized VEGFR2 normalized to VEGFR2 surface expression before stimulation. (*N* = 7 independent experiments; two-way ANOVA: ns *P* > 0.05, **P < 0.01). **g** Antibody feeding assay to assess VEGFR2 internalization in response to VEGF (3 nM) in *Ctrl* and *ROBO1/2* siRNA silenced HUVECs. Quantification of internalized VEGFR2 fluorescent intensity (right panel) (*N* = 4 independent experiments, at least 10^3^ cells analyzed per experiment; Mann–Whitney U test: *P < 0.05). **h** Golgi orientation in tip cells from *Robo1,2*^*+/+*^ and *Robo1,2*^−/−^ P5 retinas. (*N* = 3 retinas per group, at least 50 tip cells per retina were quantified, two-way ANOVA: *P < 0.05, ***P < 0.001, ns *P* > 0.05). Error bars represent mean ± s.e.m. Scale bars: **b** left panel 5 μm, **b** right panel 1 μm, **c** 2 μm, **g** 20 μm

We next tested if ROBO receptors also influenced VEGF-driven VEGFR2 endocytosis. ROBO1 was co-immunoprecipitated with VEGFR2 after SLIT2 and VEGF stimulation (Fig. 5a). SIM imaging revealed that VEGFR2, ROBO1, and ENDOA2 were clustered in close proximity at the lamellipodia in HUVEC stimulated with VEGF (Fig. 5b). These results suggest protein interaction and potential function of ROBO1 in ENDOA2-mediated endocytosis of VEGFR2. Consistent with the hypothesis, SIM analysis showed that *ROBO1/2* siRNA treatment in HUVECs abolished VEGFR2 targeting to ENDOA2 vesicles induced by VEGF (Fig. 5c, d). Consequently, *ROBO1/2* silenced cells exhibited reduced VEGFR2 internalization after VEGF treatment (Fig. 5e–g). Like *EndoA2*^−/−^ mice, retinal vascular tip cells from *Robo1*^−/−^*Robo2*^*fl/fl*^*CDH5*^*ERT2* 35^ mice exhibited impaired front-rear polarity (Fig. 5h). These results show that ROBO1/2 guides VEGFR2 toward the ENDOA2-mediated internalization pathway in response to ligand activation.

### srGAP1 mediates ROBO1–ENDOA2 interaction

**Fig. 6:**
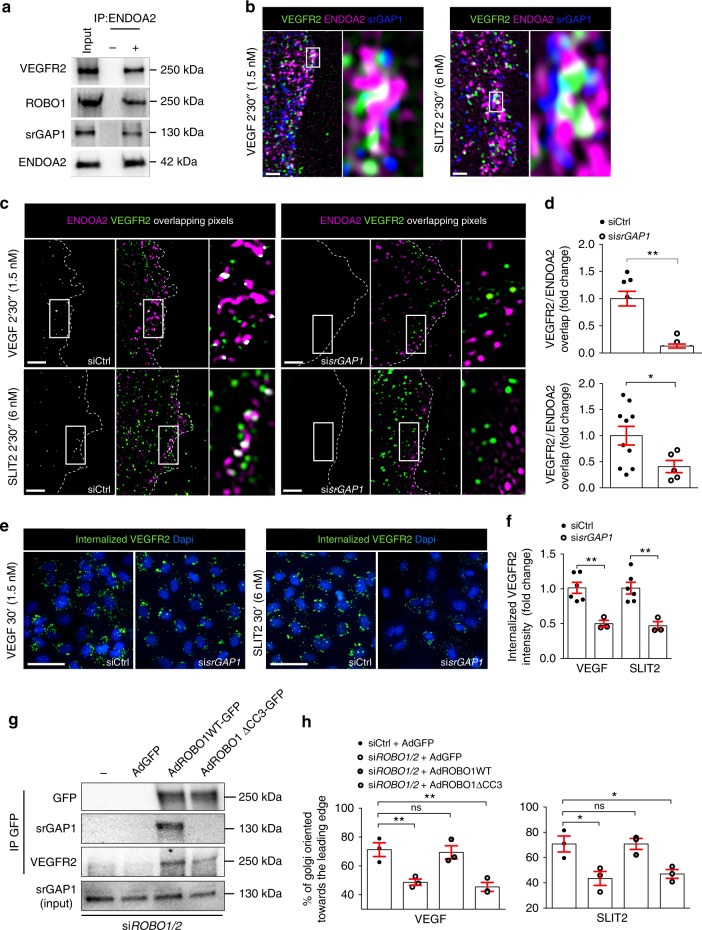
ROBO1 guides ENDOA2-mediated endocytosis via srGAP1. **a** ENDOA2 immunoprecipitation in HUVECs cultured in full medium and western-blot analysis of the indicated proteins. VEGFR2, ROBO1, srGAP1, and ENDOA2 expression from the total cell lysate are shown as loading controls (input). IP ENDOA2 (−): cell lysate incubated with beads alone; IP ENDOA2 (+): cell lysate incubated with anti-ENDOA2 antibody + beads. **b** Confocal images of HUVECs stained for VEGFR2, ENDOA2, and srGAP1 after 2′30″ VEGF (1.5 nM) or SLIT2 (3 nM) stimulation. Boxed areas are magnified to highlight VEGFR2/ENDOA2/srGAP1 pixel overlap. **c** SIM images of the lamellipodia of *Ctrl* and *srGAP1* siRNA treated HUVECs stained for ENDOA2 and VEGFR2 after VEGF or SLIT2 stimulation (1.5 or 3 nM for 2′30″). Overlapping pixels between VEGFR2/ENDOA2 fluorescent signals are shown in white. Boxed areas are magnified to highlight VEGFR2/ENDOA2 pixel overlap. **d** Quantification of pixel overlap between VEGFR2 and ENDOA2 (*N* = 10 cells per group, three independent experiments; Mann–Whitney U test: *P < 0.05, **P < 0.01). **e** Antibody feeding assay to assess VEGFR2 internalization in response to VEGF or SLIT2 (3 or 6 nM) in *Ctrl* and *srGAP1* siRNA silenced HUVECs. **f** quantification of internalized VEGFR2 fluorescent intensity (*N* = 3–6 independent experiment, at least 10^3^ cells analyzed per experiment; one-way ANOVA: **P < 0.01). **g** GFP immunoprecipitation of ROBO1/2 silenced cells transduced with the indicated constructs and western blot analysis of the indicated proteins. IP GFP (−): cell lysate from ROBO1/2 silenced cells transduced with ROBO1WT-GFP construct incubated with beads alone. **h** Golgi orientation of *ROBO1/2* silenced HUVECs transduced with the indicated constructs (*N* = 3 experiments, one-way ANOVA: ns *P* > 0.05, *P < 0.05, **P < 0.01). Error bars represent mean ± s.e.m. Scale bars: **b** 2 μm, **c** 2 μm, **e** 20 μm

To understand the molecular mechanism linking ROBO1, ENDOA2, and VEGFR2 we investigated whether SLIT ROBO GTPase-activating protein (srGAP) might constitute a physical linker between ROBO1 and ENDOA2. srGAP molecules can bind ENDOPHILINS through their SH3 domain^43^ and to the ROBO1 CC3 domain via their C-terminal region^44^. HUVECs express srGAP1 and srGAP2 (Supplementary Fig. 11a). SiRNA mediated knockdown of *srGAP1* but not *srGAP2* impaired VEGF-induced PAK activation and EC migration in response to VEGF and SLIT2 (Supplementary Fig. 11b–e). ENDOA2 could be co-immunoprecipitated with srGAP1 as well as ROBO1 and VEGFR2 in cells cultured in serum-containing medium (Fig. 6a), demonstrating interaction between these molecules. Moreover, confocal and SIM imaging showed colocalization between VEGFR2, ENDOA2, and srGAP1 at the leading edge of HUVECs treated with VEGF or SLIT2 (Fig. 6b and Supplementary Fig. 11f). siRNA silencing of *srGAP1* abolished both VEGF- and SLIT2-induced targeting of VEGFR2 to ENDOA2 positive vesicles (Fig. 6c, d) and impaired VEGF- and SLIT2-induced VEGFR2 internalization (Fig. 6e, f). Next, we reconstituted *ROBO1* and *ROBO2* siRNA treated HUVECS with adenoviral vectors encoding siRNA resistant rat full-length ROBO1-GFP (ROBO1WT) or truncated version lacking the CC3 domain GFP tagged (ROBO1ΔCC3) that binds srGAPs. Immunoprecipitation with GFP followed by immunoblotting with srGAP1 antibody showed that srGAP1 binding was strongly impaired in cells expressing ROBO1ΔCC3 (Fig. 6g), confirming that the CC3 domain is required for srGAP1 binding to ROBO1. Interestingly, VEGFR2 binding to ROBO1 was also reduced in cells expressing ROBO1ΔCC3 (Fig. 6g) demonstrating that srGAP1 promotes ROBO1-VEGFR2 complex formation. We next performed scratch wound assays with reconstituted cells and tested front-rear polarity in response to VEGF and SLIT2. Expression of ROBO1WT in *ROBO1/2* knockdown cells rescued front-rear polarity in response to both VEGF and SLIT2, whereas ROBO1ΔCC3 failed to rescue polarity (Fig. 6h). These results suggest that the physical interaction between srGAP1 and ROBO1 is required for VEGF and SLIT2-induced ENDOA2-mediated endocytosis, cell polarity and migration.

### Blocking ENDOA2 inhibits pathological angiogenesis

**Fig. 7:**
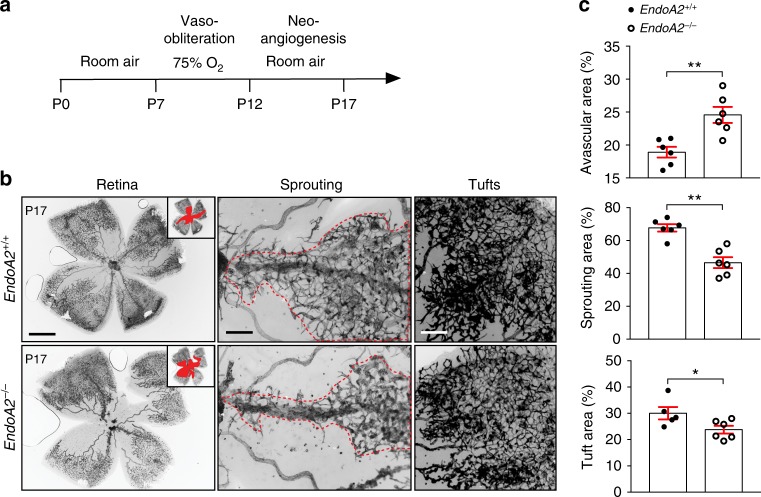
ENDOA2 promotes pathological angiogenesis. **a** Schematic of the experimental strategy to assess neoangiogenesis after oxygen-induced retinopathy (OIR). **b** Left panels show retinal flatmounts after OIR. Insets show avascular area measured for quantification. Middle panels show higher magnification of vessels sprouting from veins. Right panels show higher magnification of neovascular tufts. **c** Avascular area, sprouting and neovascular tuft quantifications of retinas shown in **b** (*N* = 6 retinas per group; Mann–Whitney U test: *P < 0.05, **P < 0.01). Error bars represent mean ± s.e.m. Scale bars: **b** left panel 1 mm, **b** middle and right panels 100 μm

To determine the function of ENDOA2 in pathological neovascularization, we subjected *EndoA2*^−/−^ mice to oxygen-induced retinopathy^45^ (Fig. 7a), which leads to pathological sprouting and formation of abnormal vascular tufts that are prone to bleeding, mimicking vision-threatening defects in infants with retinopathy of prematurity. After hyperoxia exposure, P12 pups developed vaso-obliteration leading to the formation of a capillary-free area in the center of the retina. After return to room air, hypoxia in the avascular area triggered re-growth of normal vessel sprouts form centrally located veins and the remaining capillaries in the periphery and neovascular tufts. Compared with *EndoA2*^+/+^ littermates, *EndoA2*^−/−^ mice showed decreased revascularization, measured by a significant increase of the retina avascular area, as well as decreased sprouting from veins and neovascular tuft formation (Fig. 7b, c). Thus, blockade of ENDOA2 attenuated pathological ocular neovascularization.

## Discussion

**Fig. 8:**
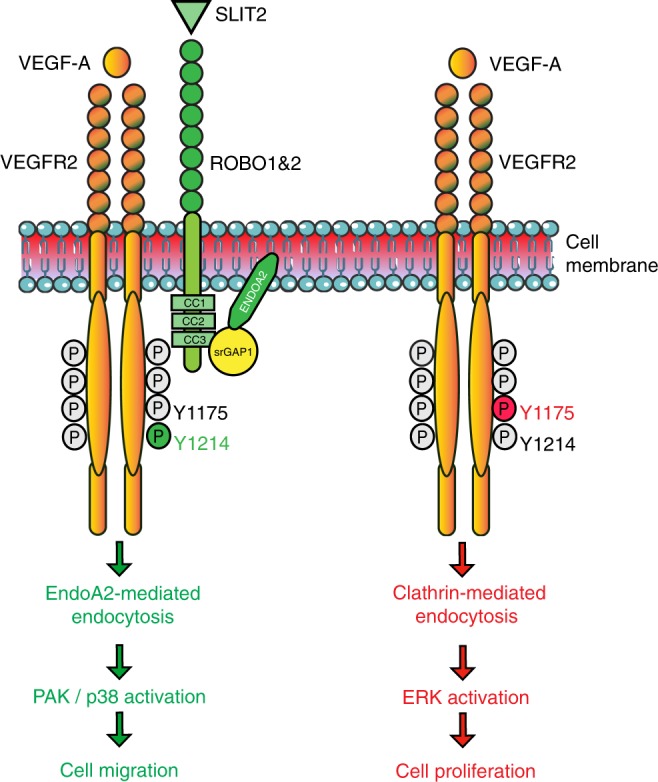
Working model for ENDOA2-mediated VEGFR2 endocytosis. ENDOA2-mediated VEGFR2 endocytosis controls EC migration independently of the CLATHRIN-mediated VEGFR2 endocytosis pathway. SLIT2-ROBO1 targets VEGFR2 to the ENDOA2-dependent internalization pathway via srGAP1, leading to downstream PAK/p38 activation and cell migration

The data reveal ENDOA2 as a regulator of polarized endothelial migration during sprouting angiogenesis (Fig. 8). Among the three ENDOPHILIN-A isoforms, only ENDOA2 was expressed in ECs and required for developmental and pathological angiogenesis. ENDOA2 promoted directional migration by regulating VEGFR2 internalization and downstream signaling to PAK, but not ERK. This result shows that distinct VEGFR2 uptake pathways can control cellular behaviors by promoting activation of select downstream signaling pathways, and reveal that VEGFR2 endocytosis via ENDOA2 promotes polarized endothelial migration.

The best studied VEGFR2 internalization route is via CME^15,16^, but VEGFR2 can be internalized by macropinocytosis or caveolea as well^46–49^. How VEGFR2 uptake via these distinct routes is regulated is poorly understood. CLATHRIN dependent VEGFR2 uptake requires the guidance molecule EPHRINB2, as shown in mice carrying endothelial *Efnb2* deletion which exhibit lack of VEGFR2 uptake and signaling^18–20^. Another guidance molecule NRP1 regulates VEGFR2 endosomal trafficking, and mice lacking endothelial *Nrp1* exhibit arteriogenesis defects because of impaired VEGF-induced ERK activation^17^. Our results identify the SLIT2-ROBO1 guidance pathway as a critical mediator of ENDOA2-mediated VEGFR2 uptake and subsequent polarized endothelial cell migration. Previous studies had shown that ENDOPHILINs are a component of CME^36,50^. In line with these findings, we find that ENDOA2 and CLATHRIN show a small overlap in unstimulated and ligand-stimulated cells. However, following VEGF stimulation, VEGFR2 segregates into distinct endosomes that are either positive for CLATHRIN or for ENDOA2, suggesting largely distinct endocytic pathways. In support of this idea, *ENDOA2* knockdown failed to affect CLATHRIN-mediated VEGFR2 endocytosis and ERK activation by VEGF, which is known to depend on CME^17^. SLIT2 stimulation preferentially drives ENDOA2 but not CLATHRIN-mediated uptake of ROBO1 and VEGFR2, thereby promoting PAK but not ERK activation in migrating ECs. Together, these findings suggest that axon guidance receptor signaling pathways in ECs function, at least in part, by guiding endocytosis of critical surface receptors such as VEGFR2. Furthermore, the data suggest that ENDOA2 and CLATHRIN-mediated VEGFR2 endocytosis are two largely independent and parallel internalization routes that trigger different downstream signaling pathways and control specific cell behaviors.

SLIT2 and VEGF treatment of ECs promoted complex formation between ROBO1 and VEGFR2 via srGAP1, leading to internalization via the ENDOA2 pathway. This process is likely facilitated by direct binding of srGAP1 to ROBO1 and ENDOA2, although the molecular details remain to be established. Association of srGAP1 and ENDOA2 was recently shown in rat brain lysates^31^, confirming data obtained here in ECs. Our study provides a mechanistic understanding for how SLIT2/ROBO1 function controls sprouting angiogenesis. We had previously shown that postnatal deletion of *ROBO1/2* and the downstream *NCK1/2* adaptors in ECs induced selective defects in front-rear polarity and angiogenic sprouting^9,35^. While ROBO1 was the predominant SLIT2-binding ROBO expressed in ECs, its deletion caused upregulation of ROBO2, which was normally expressed at very low levels, hence combined ROBO1 and 2 deletion was required to prevent SLIT2 signaling and reveal angiogenic sprouting defects in mice^35^. In vitro, deletion of *ROBO1* and *2* abolished SLIT2-induced EC front-rear polarity and migration, as expected, but also affected VEGF-induced polarity and migration^9,35^, raising the question how lack of ROBO function affected VEGF signaling mechanistically. The data shown here reveal ENDOA2-mediated endocytosis as a major pathway for ROBO-VEGFR2 interaction. We propose that ROBO1 acts as a molecular cell surface tag driving VEGFR2 toward ENDOA2 (Fig. 8). SLIT2 activation of ROBO1 was sufficient to initiate VEGFR2 internalization via ENDOA2, however, in the absence of VEGF ligand, the internalized VEGFR2 remained inactive. This explains why SLIT2 pre-treatment of ECs in vitro can reduce VEGF signaling^51,52^, because less surface receptor is available for ligand binding and signaling. In the combined presence of VEGF and SLIT2, VEGFR2 signaling toward PAK/p38 was enhanced^35,53–55^, likely via enhanced ENDOA2-mediated endocytosis. Conversely, absence of ROBO1 impaired SLIT2 and VEGF-driven ENDOA2-mediated endocytosis of VEGFR2, thereby impairing migration. Further studies are required to reveal the exact mechanism promoting the selective effects of ENDOA2-mediated endocytosis on cell polarity and migration, but the results reveal the EPA as an environment conducive for PAK/p38 activation, providing a framework for future investigations.

Our data reveal similar tip cell polarity defects in *EndoA2* and *Robo1*^*−/−*^ *Robo2*^*fl/fl*^*CDH5*^*ERT2*^ retina vessels. Interestingly, in renal glomeruli, both ROBO2 and ENDOA2 interact with NEPHRIN, a basement membrane protein regulating podocyte structure and the SLIT-diaphragm organization^50,56,57^. Mice carrying deletions of *Robo2* or *EndoA1*-*3* failed to establish a normal glomerular filtration barrier and exhibited severe proteinuria due to abnormal podocyte end-feet process formation. Thus, ENDOPHILINS may interact with the ROBO pathway in additional cell types besides ECs. Another interesting cell type to be considered are commissural neurons, that respond to SLIT midline guidance signals via ROBO1 and 2^58^. While a previous study had suggested CME as an endocytic route for ROBOs in commissural axons^59^, FEME has not been investigated yet.

The biological function of ENDOPHILINS is so far poorly explored. ENDOA1 was shown to interact with EGFR in cultured brain ECs to control cell permeability via cell junction proteins^60,61^; however, we have been unable to detect ENDOA1 expression in ECs, and single cell RNA sequencing also failed to detect *EndoA1* or *A3* in adult brain ECs^62^. In contrast to ECs, *EndoA1* and *EndoA3* are abundantly expressed in neurons^33,37,63^ and combined deletion of all *EndoA* isoform was required to induce neurological defects^36^. In line with these findings, we show that the single deletion of *EndoA2* did not affect *EndoA*1 or *A3* gene expression in ECs, or neuron number and organization in the retina. Our data reveal ENDOA2 driven endocytosis as a target to prevent pathological angiogenesis in intraocular neovascular diseases such as retinopathy of prematurity, which is characterized by excessive angiogenesis promoting vascular leak and edema, hemorrhage and retinal detachment compromising vision^64^. Specific targeting of sprouting angiogenesis could be central to therapeutic strategies in such pathologies, where anti-angiogenic approaches with VEGF blockers may produce unwanted side-effects on photoreceptor survival^65,66^.

## Methods

### Reagents and antibodies

Recombinant proteins: SLIT2 (5444-SL, R&D Systems), VEGF-A165 (293-VE, R&D Systems). Antibodies: Endophilin-A2 (1/200, sc-365704, Santa Cruz), CLATHRIN (1/400, 4796P, Cell Signaling), anti-Robo1 (MAB7118, R&D Systems), anti-GM130 (1/500, 610823, BD), anti-NG2 (1/200, AB5320, Millipore), anti-Desmin (1/200, AT3844, NovusBio), anti-VECadherin (1/200, Santa Cruz, Sc6458), anti-Collagen IV (1/300, AB769, Millipore), anti-ERG1/2/3 (1/100, SC353, Santa Cruz), anti-α-smooth muscle actin CY3 (1/200, C6198, Sigma), anti cleaved caspase-3 (9661S, Cell Signaling), anti-Podocalyxin (1/200, AF1556, R&D systems), Dapi (1/100, D1306, Life Technologies), anti-pVEGFR2 1175 (1/500, 2478, Cell Signaling), anti-pVEGFR2 1214 (1/250, 2477, Cell Signaling), anti-p44/42 MAP kinase (1/1000, phospho-ERK, 9106, Cell Signaling), anti-p44/42 MAP kinase (1/1000, total ERK, 9102, Cell Signaling), anti-pPAK1 (Thr423)/PAK2 (Thr402) (1/500, 2601S, Cell Signaling), anti-PAK1/2/3 (1/500, 2604, Cell Signaling), anti-srGAP1 (1/200, ab76926, Abcam), anti-p38 MAPK (1/500, 8690T, Cell Signaling), anti-phosp38 MAPK (1/500, 4511T, Cell Signaling), anti-pMLC2 (1/200, 3671S, Cell Signaling), anti-VEGFR2 (1/500, 9698, Cell Signaling), anti-actin (1/2000, A1978, Sigma), anti-Calretinin (1/200, MAB1568, Millipore), anti-endomucin (1/200, HM1108, Hycult), anti-GFAP (1/200, ZO334, Dako). Appropriate secondary antibodies were conjugated to horseradish peroxidase (Vector Laboratories) or fluorescently labeled (Life Technologies). IsolectinB4 (I21411), Dapi (D1306), and phalloidin^568^ were purchased from Life Technologies.

### Animals

*EndoA2*^−/−^ mice were a gift from De Camilli Lab^36^. *Robo1*^*−/−*^*Robo2*^*fl/fl*^*CDH5*^*ERT2*^ mice were previously described^35^. Mice were maintained under standard specific pathogen-free conditions. All animal procedures were reviewed and approved by the Institutional Animal Care Use Committee of Yale University and comply with all ethical regulation.

### Retina immunostaining and analysis

The eyes of P5 pups were prefixed in 4% PFA for 20 min at room temperature (RT). The retinas were dissected out and blocked during 30 min at RT in blocking buffer (1% fetal bovine serum, 3% BSA, 0.5% Triton X-100, 0.01% Na deoxycholate, 0.02% Na azide in PBS at pH 7.4). The retinas were incubated with antibodies in blocking buffer overnight. After washing with Pblec (1 mM MgCl_2_, 1 mM CaCl_2_, 0.1 mM MnCl_2_, 1% Triton X-100 in PBS), the retinas were incubated with IsolectinB4 and the corresponding secondary antibody in Pblec for 2 h at RT. Then the retinas were mounted in fluorescent mounting medium (DAKO). We performed the proliferation analysis using Click-iT EdU Alexa Fluor 647 Imaging kit (Life Technologies). P5 pups were injected with 300 μg of EdU (5 mg/ml) and euthanized 4 h later. EdU staining was done according to the manufacturer’s protocol. For soluble Flt1 binding, after dissection, retinas were blocked and permeabilzed in TNBT (100 mM Tris pH 7.4, 150 mM NaCl, 0.5% Triton X-100, 0.5% of TSA Blocking blocking reagent (FP1012, Perkin Elmer). Then, retinas were incubated in 1 μg/ml of recombinant mouse soluble Flt-1 FC chimera (471-F1-100, R&D) diluted in TNBT for 2 h at room temperature, rinsed three times in TNT (100 mM Tris pH 7.4, 150 mM NaCl, 0.5% Triton X-100), fixed with 4% PFA for 2 min at room temperature and incubated with anti-human IgG secondary antibodies diluted in TNBT overnight at 4 °C. Confocal pictures of retinas were acquired using a Leica SP5 confocal microscope with a Leica spectral detection system (Leica 15 SP detector) and the Leica application suite advanced fluorescence (LAS-AF) software. Quantification of retinal vascular development was done using the Image J software.

For immunostaining on sections, eyes were collected at P5 and fixed in 4% PFA for 1 h at room temperature. A hole was made in the cornea, and the eyes were incubated for 1 h in 10% sucrose (VWR, 27478.296) in 0.12 M phosphate buffer and then overnight at 4 °C in 30% sucrose in 0.12 M phosphate buffer. Eyes were then embedded and frozen in 0.12 M phosphate buffer containing 7.5% gelatin (Sigma, 62500) and 10% sucrose. Twenty-micrometer sections were cut with a cryostat (Leica, CM3050S). These sections were blocked in PBS containing 0.2% gelatin (VWR) and 0.25% Triton X-20 (PBS-GT) for 1 h and incubated overnight at room temperature with primary antibodies diluted in PBS-GT. Then the sections were incubated with secondary antibodies diluted in PBS-GT and 10 μg/ml Dapi.

### Embryo whole-mount immunostaining

Embryos were harvested and fixed in 4% paraformaldehyde (PFA) in PBS for 2 h at room temperature, washed with PBS and incubated for 1 h in blocking solution (1% Triton X-100, 3% bovine serum albumin (BSA, Sigma-Aldrich) and 3% heat-inactivated bovine serum (Fisher) at room temperature. Embryos were incubated with the primary antibody overnight (1:100 Endomucin (Hycult Biotech) in blocking solution. Samples were washed and incubated in secondary antibody (1:200 Goat anti-Rat AlexaFluor 488 (Invitrogen) in blocking solution) overnight. Hindbrain dissections and immunostaining were done as previously described^67^.

### Oxygen-induced retinopathy

OIR was performed as described^9,35^. Briefly, the breeding mother and P7 pups of both genders were placed in 75% O_2_ until P12. The pups were then exposed to room air for an additional 5 days until P17. Eyes were collected at P17, retinas were stained with IB4. Avascular area and vascular tufts were quantified^9,35^ using image J.

### siRNA transfection

EndophinA2 siRNA was purchased from Invitrogen (SH3GL1SS109705, 81165385; siRNAs (FlexiTube siRNA)). ROBO1 siRNA (SMARTpool: ON-TARGETplus ROBO1 siRNA L-011381-00-0005), ROBO2 siRNA (SMARTpool: ON-TARGETplus ROBO2 siRNA L-023273-01-0005), and the matching negative controls (ON-TARGETplus Non-Targeting Pool D-001810-10-05) were purchased from Dharmacon. We transfected HUVECs with 25 pmol siRNA per 6-well plate with 2.5 μl RNAiMax (Invitrogen) according to the manufacturer’s instructions. Cells were used for experiments 72 h after transfection.

### Cell culture

HUVECs were obtained from the Yale University Vascular Biology and Therapeutics Core Facility and cultured in EGM2-Bullet kit medium (CC-3156 & CC-4176, Lonza). We starved HUVECs overnight in EBM-2 supplemented with 0.1%, 0.5%, or 1% FBS before SLIT2 or VEGF treatment.

### Murine endothelial cell isolation

We harvested mouse lungs between P15 and P21, minced them and incubated them in 5 mL Dulbecco’s modified Eagle’s medium containing 2 mg/mL collagenase I (Invitrogen) for 45 min at 37 °C with shaking every 15 min followed by filtering through a 40-μm nylon mesh (BD Falcon). The cells were then centrifuged at 1000 × *g* for 5 min at 4 °C, resuspended in buffer 1 (0.1% bovine serum albumin, 2 mM EDTA, pH 7.4, in PBS), and incubated with anti-rat immunoglobulin G-coated magnetic beads (Invitrogen) precoupled with rat anti-mouse platelet/endothelial cell adhesion molecule-1 (PECAM-1; MEC13.3, BD Pharmingen, 553370) for 30 min at 4 °C in an overhead shaker. Beads were separated from the solution with a magnetic particle concentrator (Dynal MPC-S, Invitrogen). The beads were washed five times with buffer 1 and centrifuged for 5 min at 1000  × *g*, and the supernatant was removed. The purified endothelial cells were then cultured in ECGM-2 (Promocell). For western-blot analysis, lung endothelial cells (2 × 10^5^) were seeded in 60-mm dishes and cultured for 24 h in ECGM-2 at 37 °C and 5% CO_2_.

### Western blot

Cells were lysed in lysis buffer including phosphatase and protease inhibitors (Thermo Scientific, 78420, 1862209). Equal amounts of proteins were separated on 4–15% Criterion precast gel (#567-1084, Bio-Rad) and transferred on nitrocellulose membrane (Bio-Rad). Western blots were developed with chemiluminescence HRP substrate (Millipore, WBKLS0500) on a Luminescent image analyzer, ImageQuant LAS 4000 mini (Ge Healthcare). See Supplementary Figs. 12 and 13 for the uncropped immunoblots.

### Immunoprecipitation

Cell lysates were prepared in 50 mM Tris-HCl at pH 7.4, 50 mM NaCl, 0.5% Triton X-100, phosphatase and protease inhibitors, centrifuged at 16,000 × *g* for 20 min. Protein concentration was quantified using Bradford assay (Pierce). In total, 500 μg of protein from cell lysate were incubated overnight at 4 °C with 10 μg/ml of anti-ENDOA2 (Santa Cruz) or anti-GFP antibodies (BD Pharmingen), and finally incubated with protein A/G magnetic beads (88802, Thermo Scientific) for 2 h at 4 °C. The immunocomplexes were washed three times in lysis buffer and resuspended in 2X Laemmli’s sample buffer. For western-blot analysis, 50 μg of protein was loaded for each condition.

### Cell immunostaining

Cells were plated on gelatin coated glass bottom dishes. Growing cells were fixed for 10 min with 4% paraformaldehyde (PFA) and permeabilized with 0.1% Triton X‐100, for 10 min prior to overnight incubation with primary antibody and then secondary antibody conjugated with fluorophore. Samples were mounted with ProLong Gold (Invitrogen).

### SIM

Images were acquired using a U-PLANAPO 603/1.42 PSF, oil immersion objective lens (Olympus, Center Valley, PA) and CoolSNAP HQ2 CCD cameras with a pixel size of 0.080 mm (Photometrics, Tucson, AZ) on the OMX version 3 system (Applied Precision) equipped with 488, 561, and 642 nm solid-state lasers (Coherent and MPB communications). Samples were illuminated by a coherent scrambled laser light source that had passed through a diffraction grating to generate the structured illumination by interference of light orders in the image plane to create a 3D sinusoidal pattern, with lateral stripes ~0.270 nm apart. The pattern was shifted laterally through five phases and through three angular rotations of 60 for each z section, separated by 0.125 nm. Exposure times were typically between 50 and 200 ms, and the power of each laser was adjusted to achieve optimal intensities of between 2000 and 4000 counts in a raw image of 16-bit dynamic range, at the lowest possible laser power to minimize photo bleaching. Raw images were processed and reconstructed to reveal structures with 100–125 nm resolution^68^. The channels were then aligned in x and y, and rotationally using predetermined shifts as measured using a target lens and the Soft-worx alignment tool (Applied Precision). RG2B Colocalization plugin for Image J was used to isolate colocalized pixel data with automatic selection threshold values and express the data as the average of the corresponding red and green channels.

### Biotinylation

HUVECs were grown to confluence and starved overnight in EBM2 with 0.5% FBS. Cells were rinsed, incubated with EZ-Link Sulfo-NHS-SS-Biotin (0.25 mg/ml, Thermo Scientific) at 4 °C for 1 h in PBS and rinsed with 50 mM glycine in PBS to stop the reaction. A portion of the cells were harvested and used to determine total biotinylated cell surface protein. The remaining cells were rinsed once with cold media +1% BSA, stimulated with EBM2 containing VEGF (25 ng/ml or 1.5 mM) or SLIT2 (1 μg/ml or 6 mM) at 37 °C for different times and then rinsed and incubated twice for 20 min each time on ice with the membrane-nonpermeable reducing agent GSH (45 mM, Sigma) in 75 mM NaCl, 75 mM NaOH, 1 mM EDTA, 1% BSA. GSH was quenched by incubating twice for 5 min each time with iodoacetamide (5 mg/ml) in PBS. Cell lysates were prepared using NP-40 lysis buffer (Roche). In total, 200 μg of protein from the cell lysate was immunoprecipitated with 50 μl of NeutrAvidin beads (Invitrogen) at 4 °C overnight, after which the beads were rinsed and resuspended in Laemmli SDS sample buffer. Samples were analyzed by SDS–PAGE followed by western blotting with anti-VEGFR2 antibody. The remaining cell lysates after bead incubation were used to blot VEGFR2, ROBO1, ENDOA2, and ACTIN loading controls.

### Antibody feeding assay

HUVECs were grown to confluence and starved overnight in EBM2 with 0.5% FBS. Cells were rinsed, incubated with anti-VEGFR2 antibody (AF357, R&D) at 4 °C for 20 min in EBM2 containing 0.5% FBS and rinsed with cold PBS. Cells were then incubated with EBM2 containing VEGF (25 ng/ml or 1.5 mM) or SLIT2 (1 μg/ml or 6 mM) at 37 °C for 30 min. Cells were rinsed twice for 2 min with cold PBS pH 2.5. After fixation with 4% PFA for 10 min at room temperature and permeabilization with 0.1% triton/PBS, cells were incubated with anti-Goat alexa^488^ antibody in 1% BSA/PBS for 1 h at room temperature.

### Scratch wound migration

We grew confluent monolayer of HUVECs in 6-well plates. Twenty-four hours after siRNA transfection, we starved the cells for 18 h in EBM-2 medium with 1% FBS. We created a horizontal wound in the confluent monolayer using a sterile 200-μl pipette tip. Next, we incubated the cells in EBM-2 supplemented with VEGF-A (50 ng/ml) or SLIT2 (1 μg/ml) at 37 °C for 18 h. Pictures of scratch wounds were taken just before stimulation (time 0) and after 18 h. We calculated the extent of cell migration using ImageJ software.

### Cell proliferation assay

The xCELLigence RTCA DP analyzer was used to measure proliferation of control and *ENDOA2* knockout HUVEC (10,000 cells/well) in response to VEGF-A (100 ng/ml, 6 nM). The plate was monitored every 15 min for 48 h.

### Apoptosis analysis

The in vitro apoptosis analysis was performed using cleaved caspase-3 staining of confluent HUVEC monolayers. Twenty-four hours after siRNA transfection, the confluent cells were starved during 24 h (EBM2, 0.5% FBS).

### Microarray

Data were extracted from a previous study^69^.

### qPCR

RNAs from HUVEC or from MLECs were purified using RNeasy-kit (Qiagen). One microgram RNA was reverse transcribed using IScript cDNA Synthesis Kit (Bio-Rad) and quantitative PCR were assayed (15 ng cDNA) using SYBR Green Supermix (Bio-Rad) and the corresponding primers: mouse *EndoA1* (forward: (CGGATGAGCCTAGAGTTTGC; reverse: GCTGATCCATTTGGACACCT); mouse *EndoA2* (forward: TCCTTCGGCACCACTTATT; reverse: CGGTGTTCAGCATAGTCAGC); mouse *EndoA3* (forward: GGCTCAAGAAGCAGTTCCAC; reverse: GTGGATGTCACCAGCAAG); mouse *Vegfa* (QT00160769, Qiagen); mouse Slit2 (QT00163828); mouse *Actb* (QT01136772); Human *ENDOA1* (QT00012796, Qiagen); human *ENDOA2* (QT00016415, Qiagen); human *ENDOA3* (QT00041027, Qiagen); human *ACTB* (QT01680476, Qiagen). The data were first normalized to actin level in each sample, and the relative expression levels of different genes were calculated by the comparative Ct method^70^.

### Statistical analysis

For continuous variables, data are presented as mean ± SEM. Between-group comparisons used the Mann–Whitney U test or *t*-test depending on the sample size for continuous variables. In cases more than two groups are compared one-way or two-way ANOVA test were performed as appropriate, followed by Turkey’s multiple comparison or Bonferroni multiple comparison tests, respectively, if *P* < 0.05. A value of *P* < 0.05 was considered statistically significant. All the analyses were performed using Prism 7.0 software (GraphPad).

### Reporting summary

Further information on research design is available in the Nature Research Reporting Summary linked to this article.

## Supplementary information

Supplementary information

Reporting Summary

## Data Availability

All data that support the findings of this study are available within the article and its Supplementary Information File and from the corresponding author upon reasonable request.
