# Supplementary material for: Endophilin-A2 dependent VEGFR2 endocytosis promotes sprouting angiogenesis
Source: Nat Commun. 2019 May 28;10:2350. doi: 10.1038/s41467-019-10359-x (PMC6538628; doi:10.1038/s41467-019-10359-x)
Supplement: Supplementary file 1 — Supplementary information [file 41467_2019_10359_MOESM1_ESM.pdf]

EndophilinA2 dependent VEGFR2 endocytosis promotes sprouting angiogenesis

Genet et al.

Supplementary Figure 1

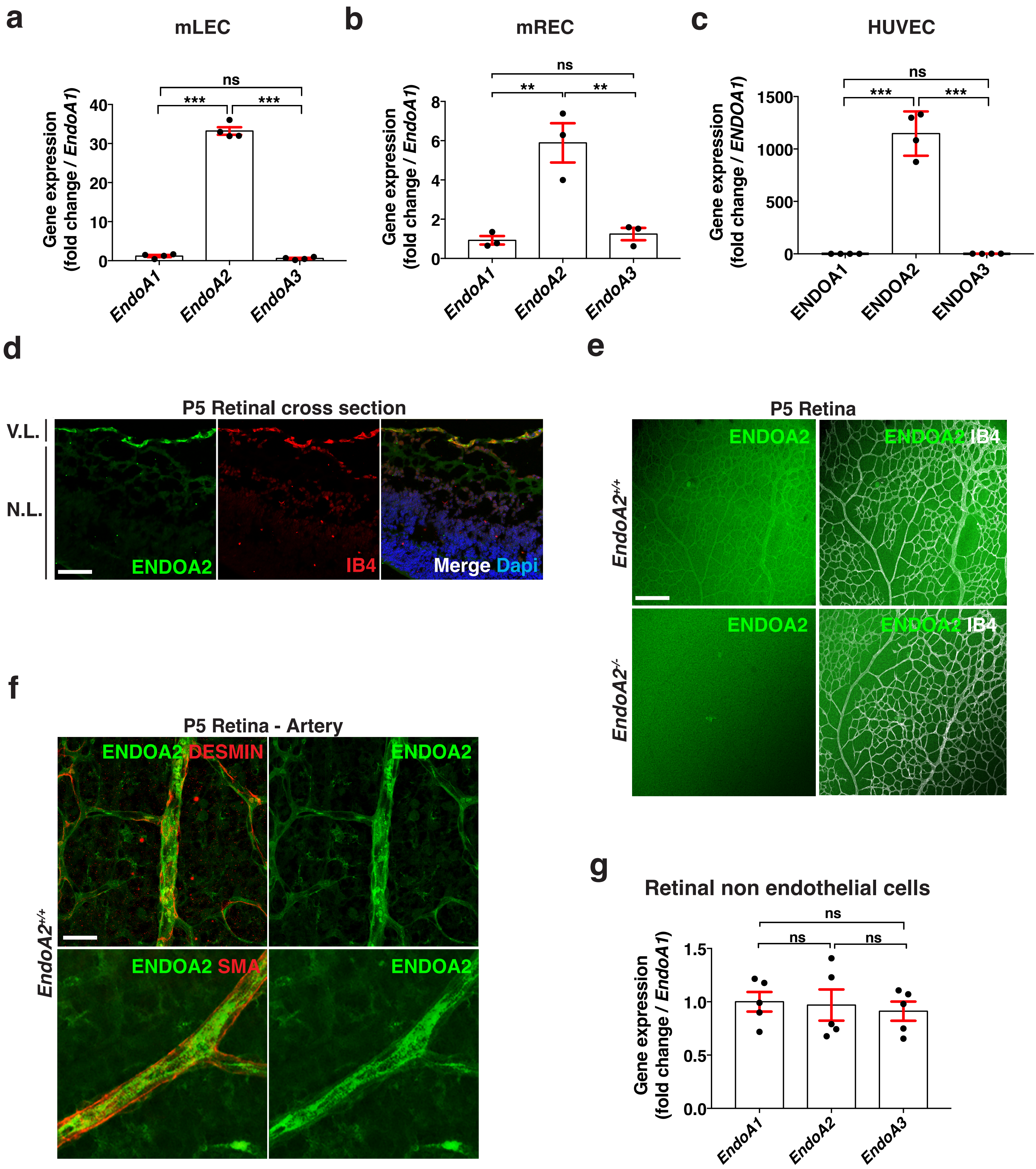

Supplementary Fig.1. ENDOPHILINs expressions in ECs

a, Gene expression of *EndoA1, A2* and *A3* in mouse lung EC (mLEC) from *EndoA2*$^{+/+}$ mice analyzed by qPCR. Gene expression is compared to *EndoA1* gene expression, and actin was used as housekeeping gene (*N*=4 WT mice; one-way ANOVA: ns P>0.05, ***P<0.001). **b,** Gene expression of *EndoA1, A2* and *A3* gene level expression in EC from P5 WT mice retina (mREC) analyzed by microarray analysis. Gene expression is compared to *EndoA1* gene expression (*N*=3 mice; one-way ANOVA, **P<0.01). **c,** Gene expression of *ENDOA1, A2* and *A3* in HUVEC analyzed by qPCR. Gene expression is compared to *EndoA1* gene expression, actin was used as housekeeping gene (*N*=4 independent experiments; one-way ANOVA: ns P>0.05, ***P<0.001). **d,** ENDOA2, IB4 and DAPI staining in a retinal cross-section from P5 WT mouse shows ENDOA2 expression in the vascular layer. V.L.: vascular layer; N.L.: neuronal layer. **e,** ENDO2 and IB4 staining in flat-mounted retinas from P5 *EndoA2*$^{+/+}$ and *EndoA2*$^{-/-}$ mice. **f,** ENDO2 and DESMIN (upper panel) or ENDOA2 and SMA (lower panel) staining in flat-mounted retinas from P5 *EndoA2*$^{+/+}$ show that pericytes or smooth muscle cells express ENDOA2. **g,** Gene expression of *EndoA1*, *A2* and *A3* gene expression in retinal non ECs from P5 WT mice retina measured by microarray analysis (*N*=5 mice; one-way ANOVA: ns P>0.05). Error bars represent mean±s.e.m. Scale bars: d: 200µm; e: 500µm; f: 40µm.

Supplementary Figure 2

a

**Genotype**	*EndoA2*$^{+/+}$	*EndoA2*$^{+/-}$	*EndoA2*$^{-/-}$
**Observed / Expected**	*67 / 49,5 mice*	*84 / 99 mice*	*47 / 49.5 mice*

b

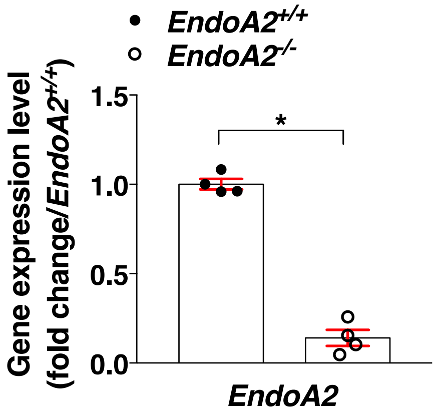

c

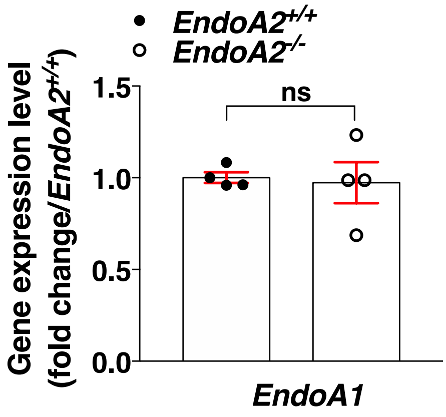

d

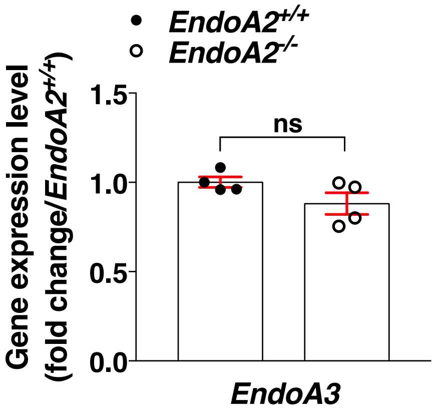

e

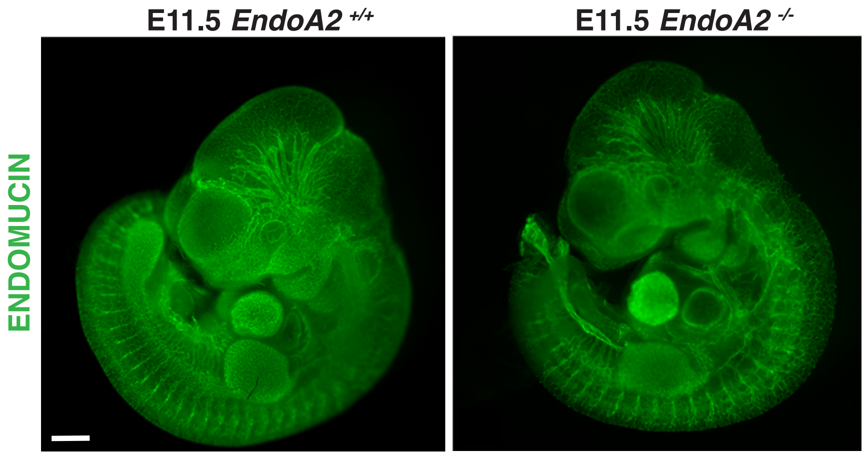

f

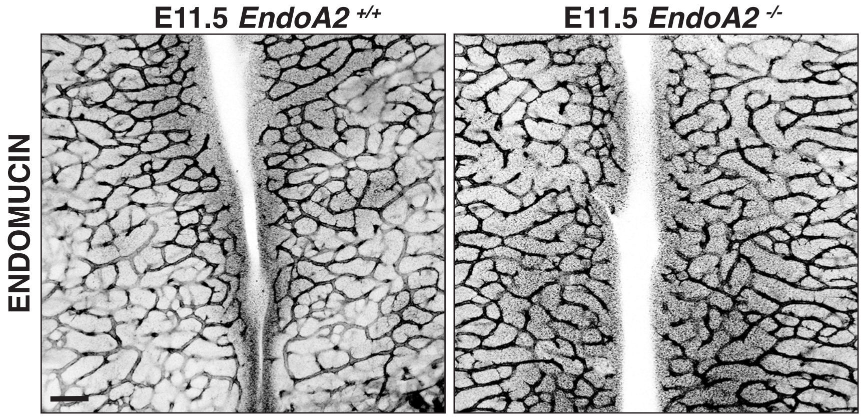

g

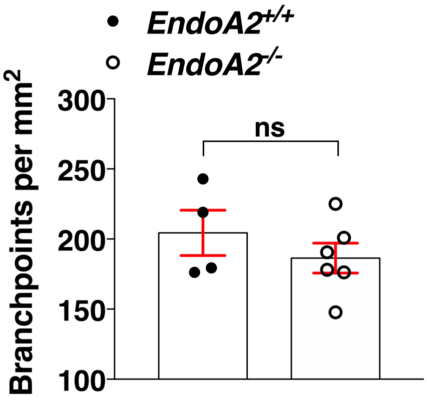

Supplementary Fig. 2. ***Embryonic development of EndoA2$^{-/-}$ mice.***

a, Number of *EndoA2*$^{+/+}$*, EndoA2*$^{+/-}$ and *EndoA2*$^{-/-}$ littermate mice at P5. 198 mice from 23 litters were analysed. **b-d,** Gene expression of *Endo A1* (b), *A2* (c) and *A3* (d) in mLEC from *EndoA2*$^{+/+}$ and *EndoA2*$^{-/-}$ mice analyzed by qPCR. *Actin* was used as housekeeping gene. (*N*=4 mice; Mann- Whitney U test: ns P>0.05, *P<0.05). **e,** Endomucin staining of E11.5 embryos. **f,** Endomucin staining of E11.5 embryo hindbrains. **g**, Quantification of the branchpoint number of the hindbrain vasculature (*N*=2 to 4 hindbrains; Mann-Whitney U test: ns P>0.05). Error bars represent mean±s.e.m. Scale bars: e: 500µm; f: 100µm.

Supplementary Figure 3

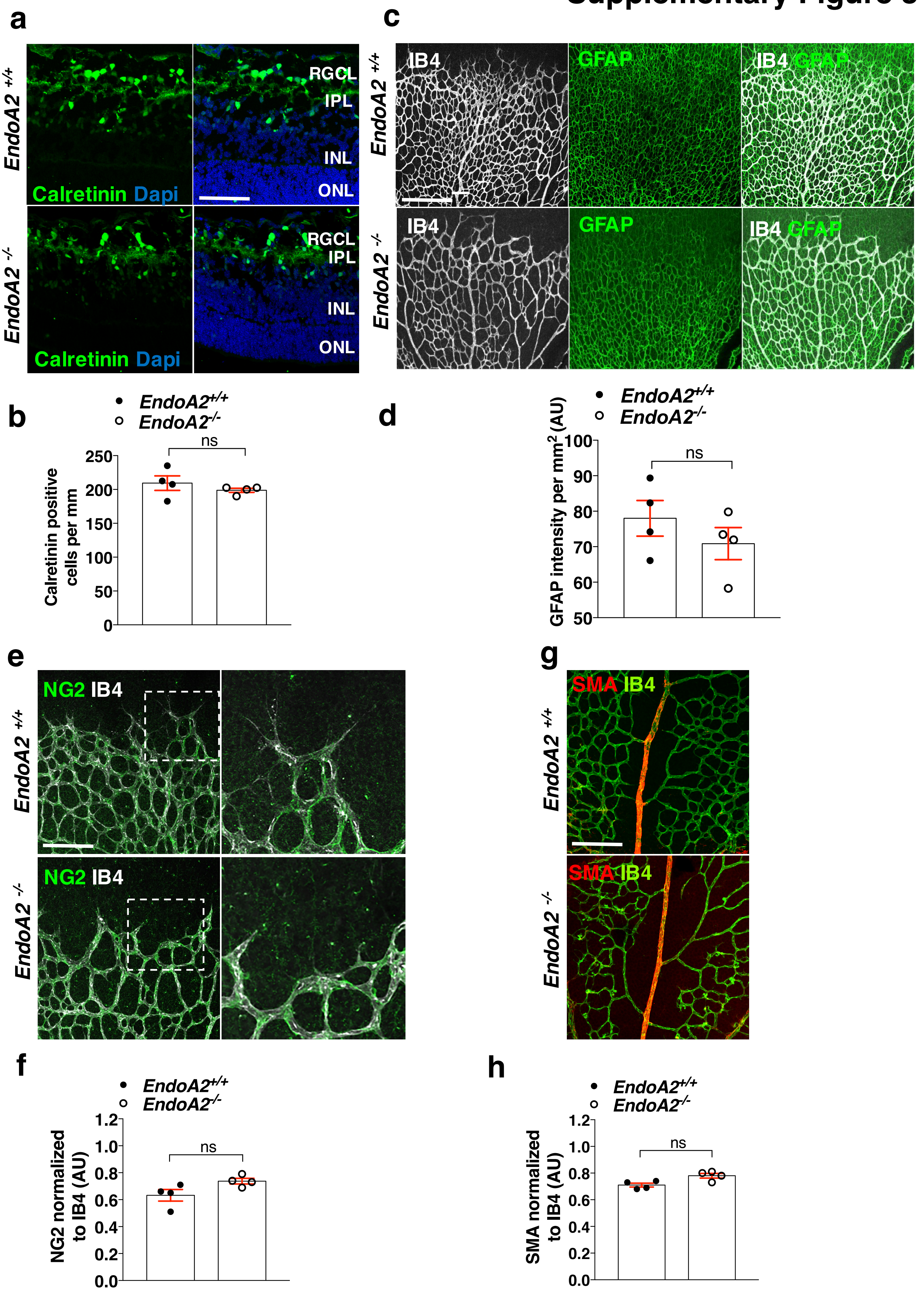

Supplementary Fig. 3. Neural organization and mural cells coverage.
a, Calretinin and Dapi staining in retinal cross sections from P5 mice. RGCL: Retinal ganglion cell layer, IPL: inner plexiform layer, INL: inner nuclear layer, ONL: outer nuclear layer. **b,** Quantification of calretinin positive cells (*N*=4 retinas; Mann-Whitney U test: ns P>0.05). **c,** GFAP and IB4 staining of P5 *EndoA2*$^{+/+}$ and *EndoA2*$^{-/-}$ retinas. **d,** Quantification of GFAP intensity (*N*=4 retinas; Mann-Whitney U test: ns P>0.05. **e**, NG2+ pericyte coverage of IB4+ vessels in P5 mice. Magnification of the boxed areas shown on the right. **f,** Quantification of NG2 intensity normalized to IB4 intensity (*N*=4 retinas; Mann-Whitney U test: ns P>0.05). **g,** SMA staining in P5 mice. **h,** Quantification of SMA intensity normalized to IB4 intensity (*N*=4 retinas; Mann-Whitney U test: ns P>0.05). Error bars represent mean±s.e.m. Scale bars: a, 50µm; c, 500µm; e, 100µm; g, 100µm.

Supplementary Figure 4

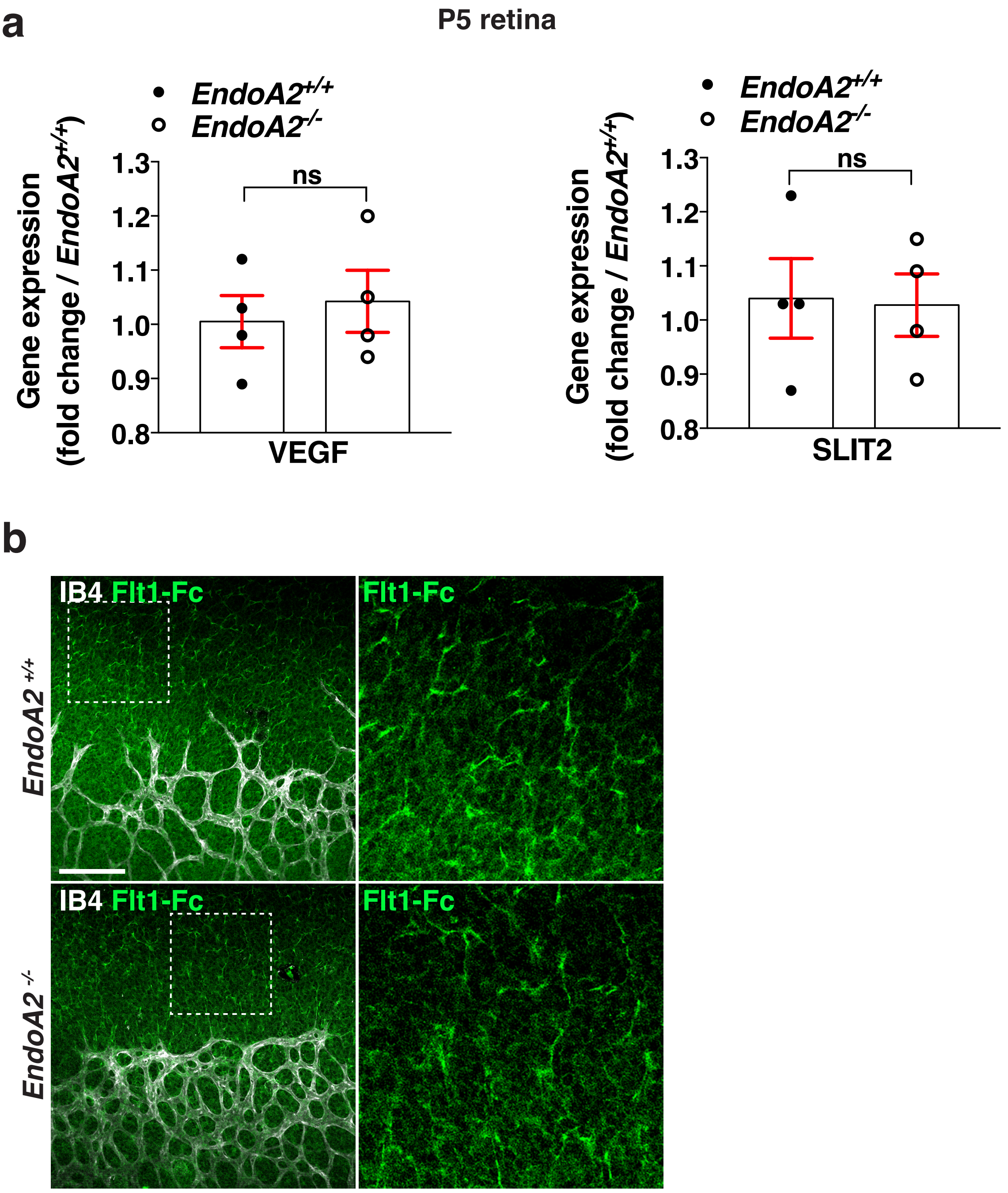

Supplementary Fig. 4. Vascular growth factors expression.
a, Gene expression of *Vegf* and *Slit2* in retinas from *EndoA2*$^{+/+}$ and *EndoA2*$^{-/-}$ P5 mice analyzed by qPCR. *Actin* was used as housekeeping gene. (*N*=4 retinas; Mann-Whitney U test: ns P>0.05). **b,** VEGF labelling in retinas from *EndoA2*$^{+/-}$ and *EndoA2*$^{-/-}$ P5 mice using Flt1-Fc chimera protein and Alexa fluor-488 anti-human secondary antibody. Vasculature is stained using IB4. Error bars represent mean±s.e.m. Scale bars: b, 100µm.

Supplementary Figure 5

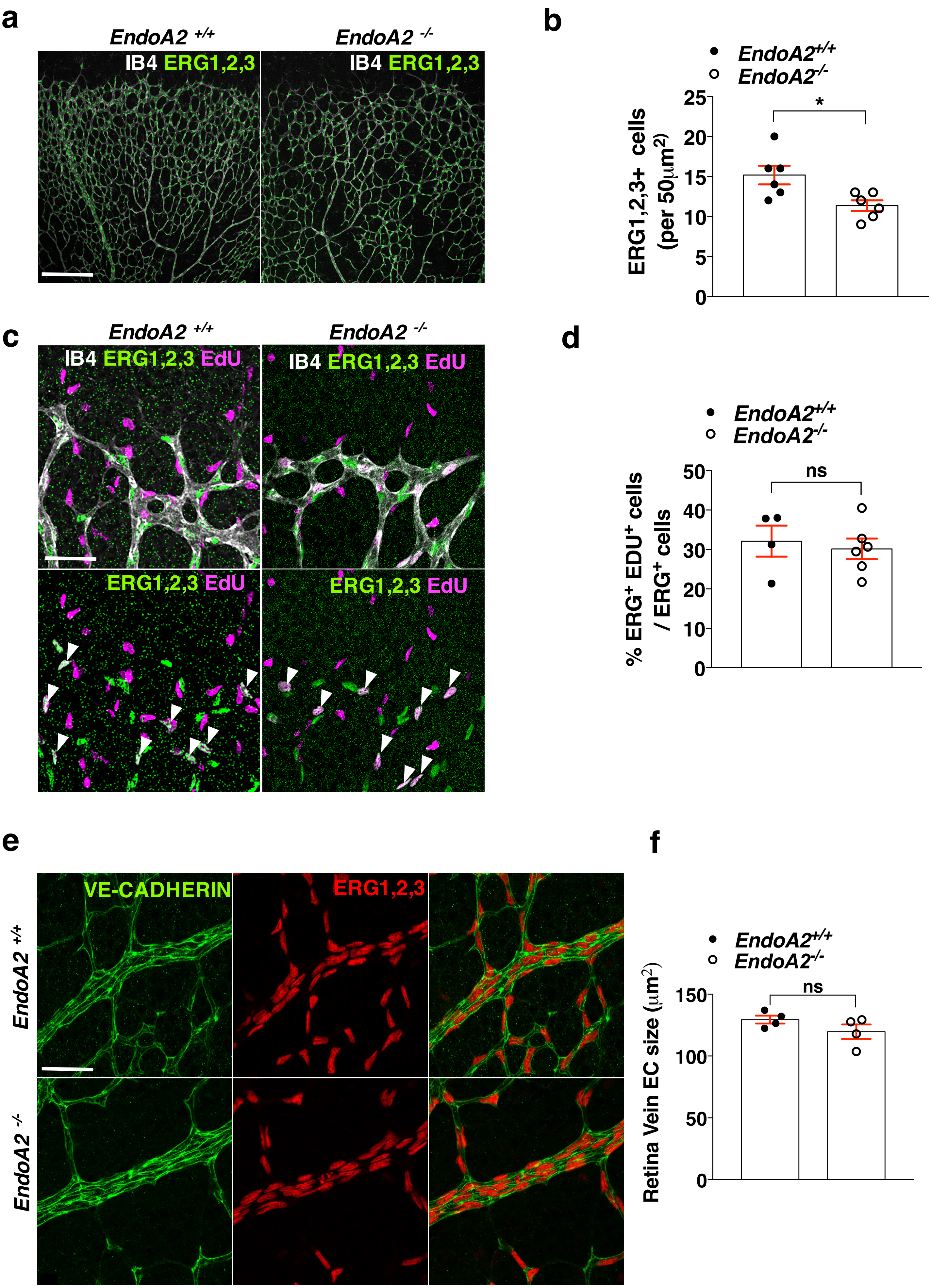

Supplementary Fig. 5. *EndoA2* deletion does not affect EC proliferation.

a, Labelling of ERG1,2,3 and IB4 of retinas from P5 control and *EndoA2* $^{-/-}$ mice. **b**, Quantification of the number of ERG1,2,3 positive cells (*N*=6 retinas; Mann-Whitney U test: * P<0.05). **c,** Labeling for ERG1,2,3, EdU and IB4 in the sprouting front of retinas from P5 control and EndoA2$^{-/-}$ mice. Mice received a 3h EdU pulse before collecting retinas. White arrowheads indicate ERG1,2,3 and EdU double positive cells. **d**, Quantification of the number of ERG1,2,3 and EdU double positive cells normalized to the total number of ERG1,2,3 positive cells (*N*=4 to 6 retinas; Mann-Whitney U test: ns P>0.05) **e,** VE-CADHERIN and ERG1,2,3 staining of P5 control and *EndoA2*$^{-/-}$ mice. **f**, Quantification of vein EC size (*N*=4 retinas; Mann-Whitney U test: ns P>0.05). Error bars represent mean±s.e.m. Scale bars: a, 500µm; c, 50µm; e, 40µm.

Supplementary Figure 6

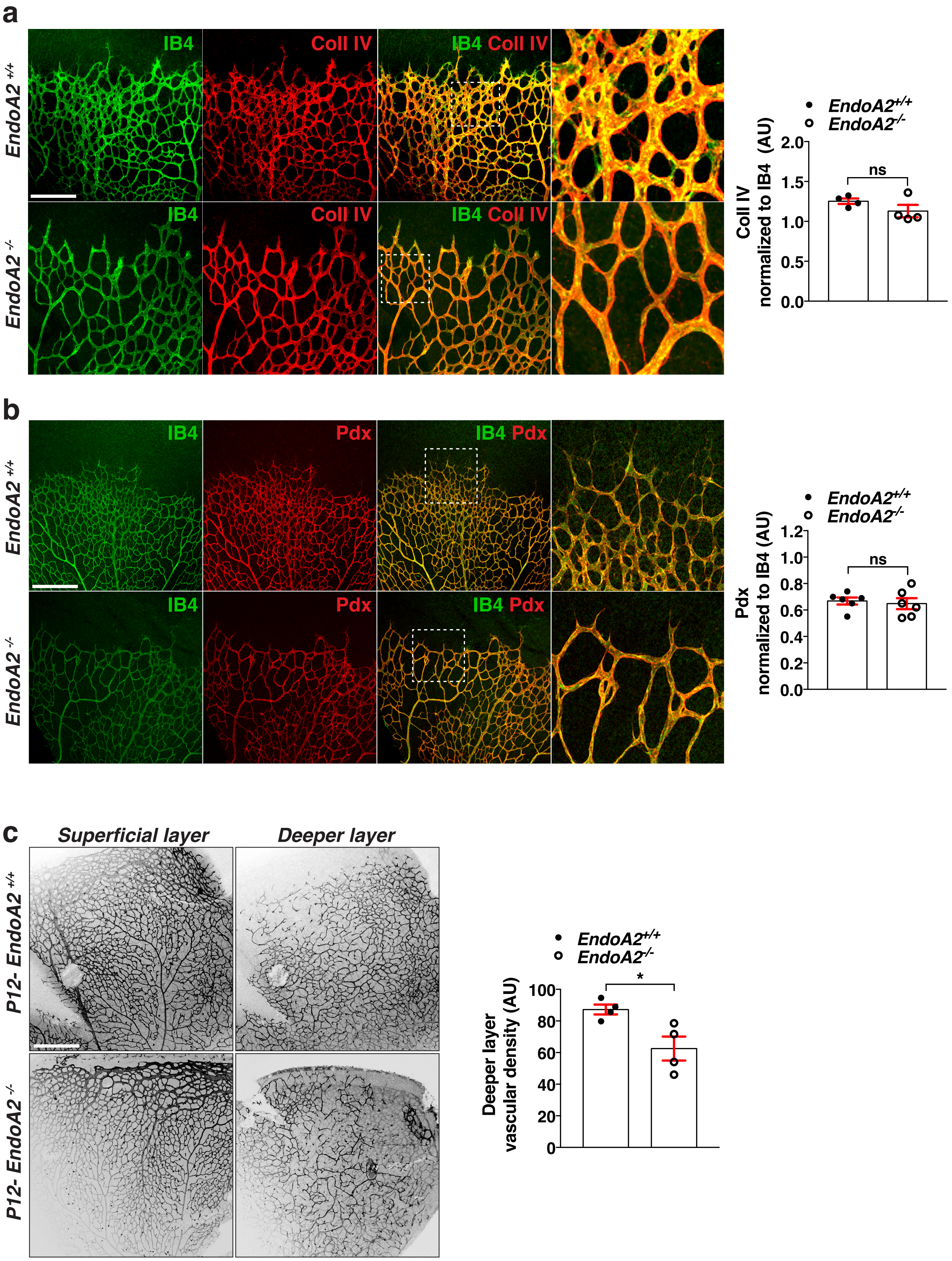

Supplementary Fig.6. Characterization of P5 and P12 *EndoA2*$^{-/-}$ retinas.

a, Collagen IV (Coll IV) staining of basement membrane coverage in retinas of mice with the indicated genotypes. Insets show magnification of the dashed area of the corresponding images. Right panel shows quantification of Coll IV+ intensity normalized to IB4 intensity (*N*=4 retinas; Mann-Whitney U test: ns P>0.05). **b,** IB4/Podocalyxin (Pdx) staining of P5 retinas from *EndoA2*$^{+/+}$ and *EndoA2*$^{-/-}$ mice. Insets show magnification of the dashed area of the corresponding images. Right panel shows quantification of Pdx intensity normalized to IB4 intensity (*N*=6 retinas; Mann-Whitney U test: ns P>0.05). **c,** IB4 stained P12 retinal flat mounts of *EndoA2*$^{+/+}$ and *EndoA2*$^{-/-}$ mice. Superficial and deeper vascular layer are shown. Right panel shows quantification of the deeper layer vascular density (*N*=4 retinas; Mann-Whitney U test: *P<0.05). Error bars represent mean±s.e.m. Scale bars: a and b, 200μm; c, 500μm.

Supplementary Figure 7

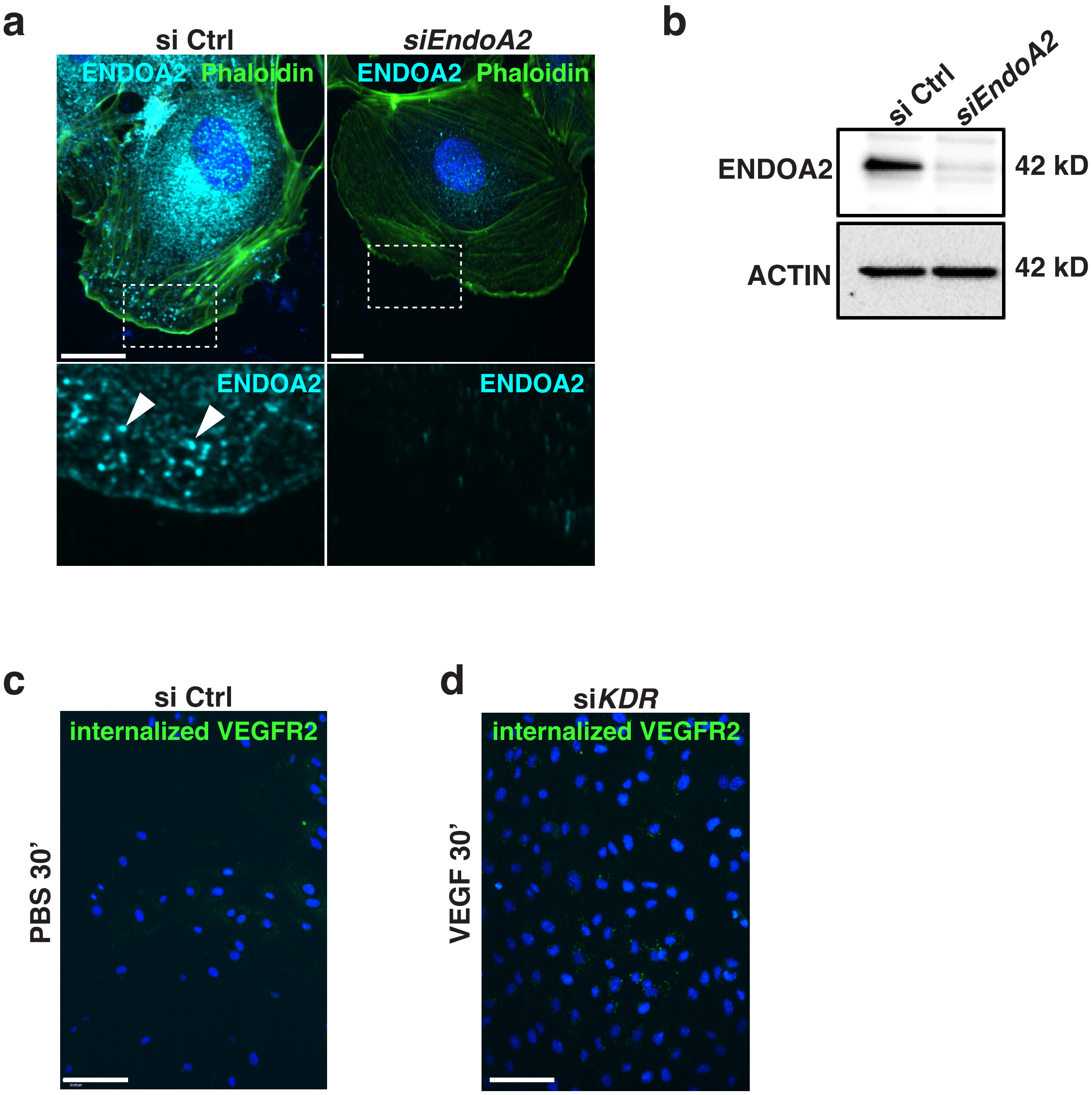

Supplementary Fig.7. ENDOA2 expression in HUVEC.

a, ENDOA2 and Phalloidin staining in Ctrl and *ENDOA2* siRNA silenced HUVEC. **b,** ENDOA2 expression in Ctrl and *ENDOA2* siRNA silenced HUVEC analyzed by Western blot. **c-d** Specificity controls for experiments shown in main Fig 2c. Antibody feeding assay of VEGFR2 internalization in response to PBS in Ctrl HUVECs (c) or in response to VEGF in VEGFR2 siRNA silenced HUVECs (d). Note the absence of internalized VEGFR2 staining in both conditions.

Supplementary Figure 8

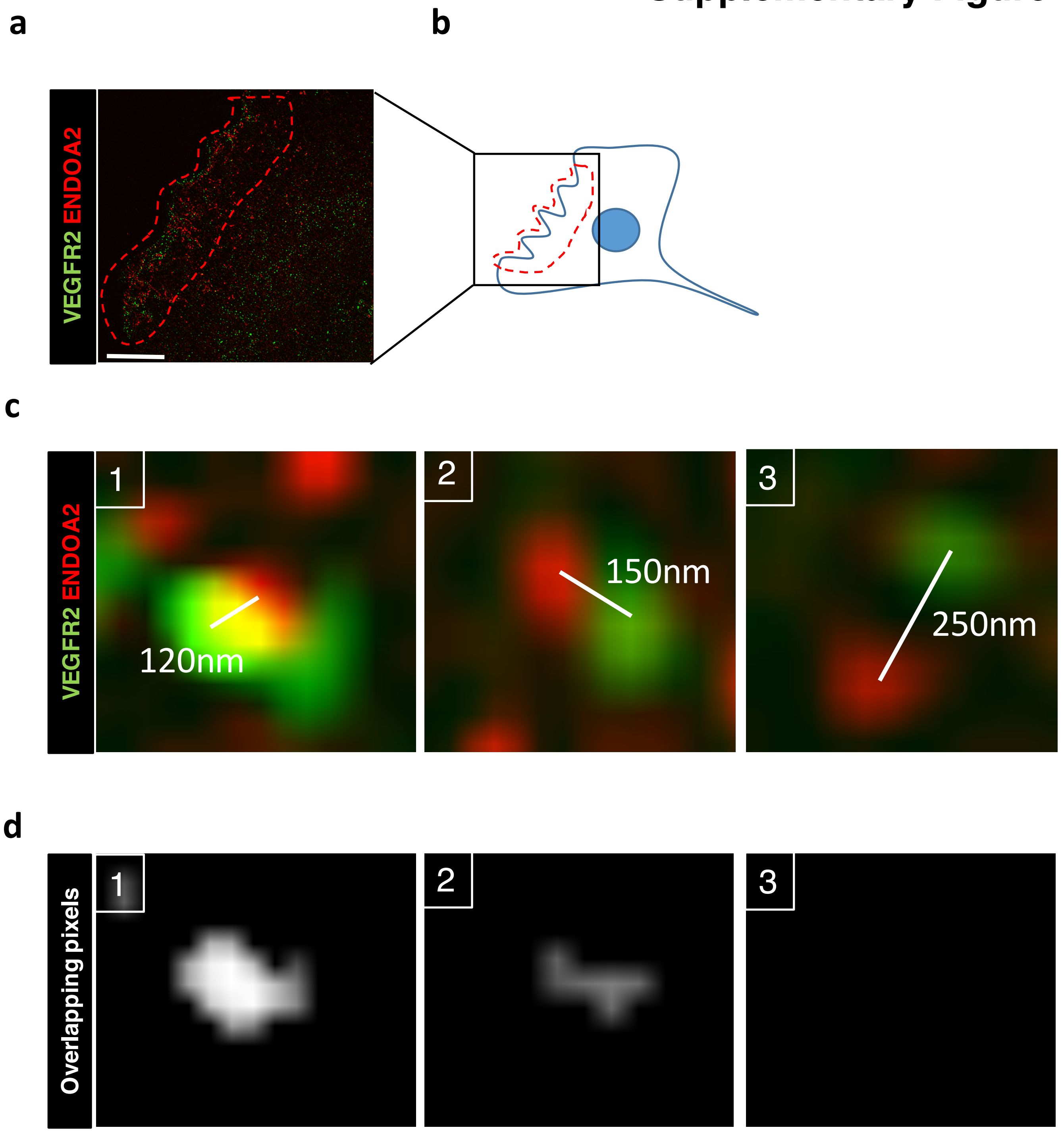

Supplementary Fig.8. Method to quantify SIM signals.

a, All SIM images were acquired at the lamellipodia of sparse HUVECs after 2min 30 VEGF (1.5nM) stimulation. **b**, Cartoon indicating the position of a representative SIM image within the cell. The red dashed shape outlines the area where VEGFR2 and ENDOA2 overlapping pixels were analyzed and quantified. **c,** Three representative images of VEGFR2 and ENDOA2 stainings. White lines indicate the distance between the center of the green (VEGFR2) and red (ENDOA2) signals. No overlapping pixels were detected for a distance greater than 200nm between the center of the green and the red signal (inset 3). **d**: Pixel overlap measured by Image J sofware (RG2B Colocalization plugin) of the corresponding pictures showed in (**c**). Scale bars: a, 3μm.

Supplementary Figure 9

a

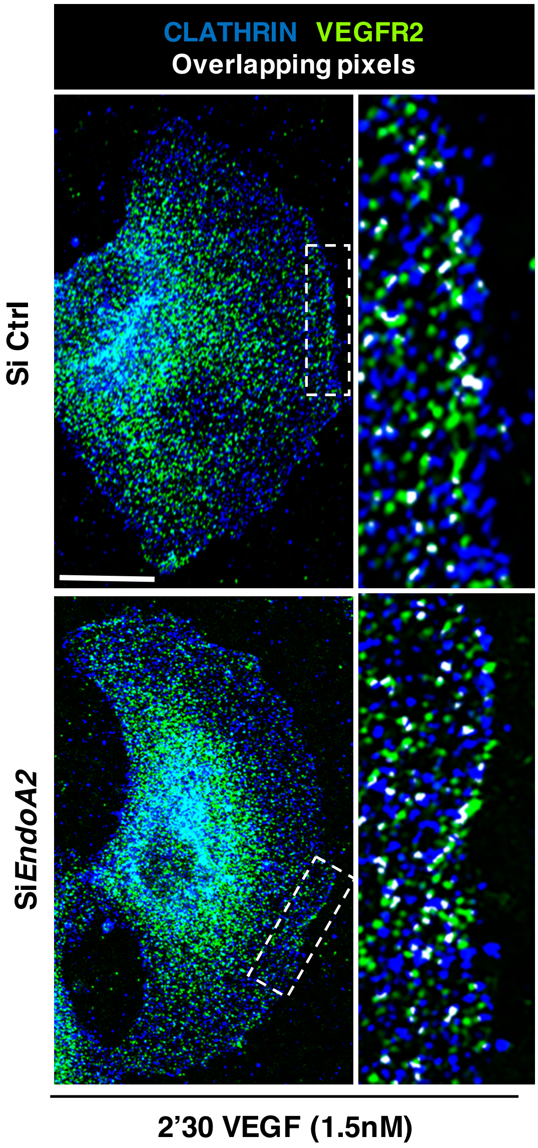

b

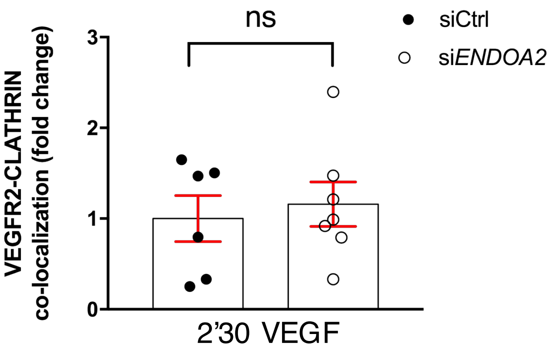

Supplementary Fig.9. *EndoA2* deletion does not affect clathrin mediated endocytosis.
a, SIM images of Ctrl and *EndoA2* siRNA treated HUVECs stained for CLATHRIN and VEGFR2 after VEGF stimulation (1.5nM for 2min30). Overlapping pixels are shown in white in the magnified boxed areas on the right. **b,** Quantification of the percentage of overlap between VEGFR2/CLATHRIN fluorescent signals (*N*=6 cells per group analyzed from 2 independent experiments; Mann-Whitney U test: ns P>0.05). Error bars represent mean±s.e.m. Scale bars: **a**, 2μm.

Supplementary Figure 10

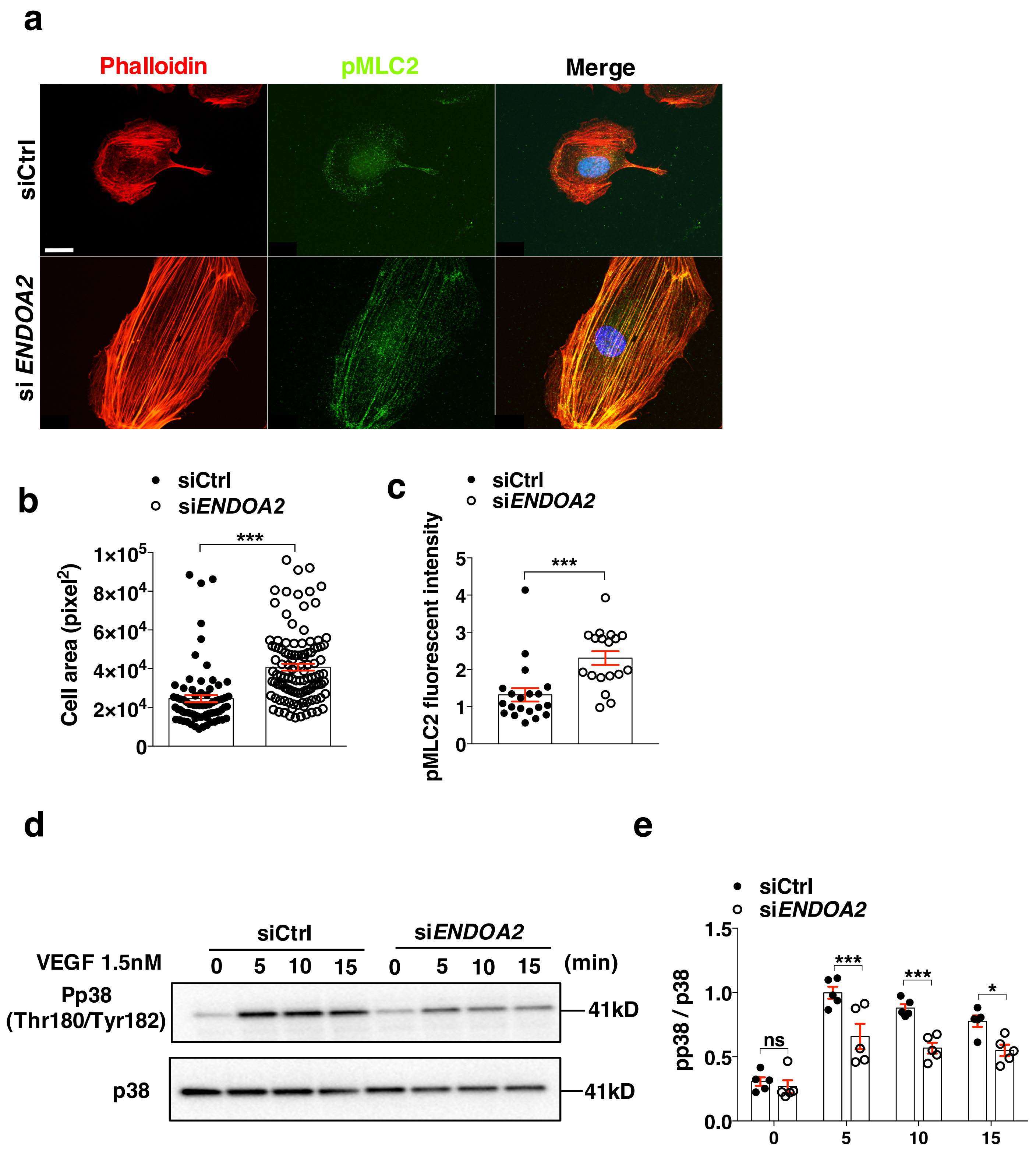

Supplementary Fig.10. *EndoA2* deletion affects EC migration.
a, Phalloidin and phospho-myosin light chain 2 (pMLC2) staining of HUVEC transfected with Ctrl or *ENDOA2* siRNA. **b,** Quantification of cell area of HUVEC transfected with the indicated siRNAs (at least 50 cells analyzed per condition; t-test: ***P<0.001). **c,** Quantification of pMLC2 intensity in HUVEC transfected with Ctrl or ENDOA2 siRNA (18 to 20 cells analyzed per condition; t-test: ***P<0.001). **d**, Western-blot analysis of VEGF-induced (1.5nM) phospho-p38 in *Ctrl* and *ENDOA2* siRNA silenced HUVECs. **e,** Quantification of p38 phosphorylation normalized to total p38 (*N*=5 independent experiments; two-way ANOVA: ns P>0.05, *P<0.05, ***P<0.001). Error bars represent mean±s.e.m. Scale bars: a, 10μm.

Supplementary Figure 11

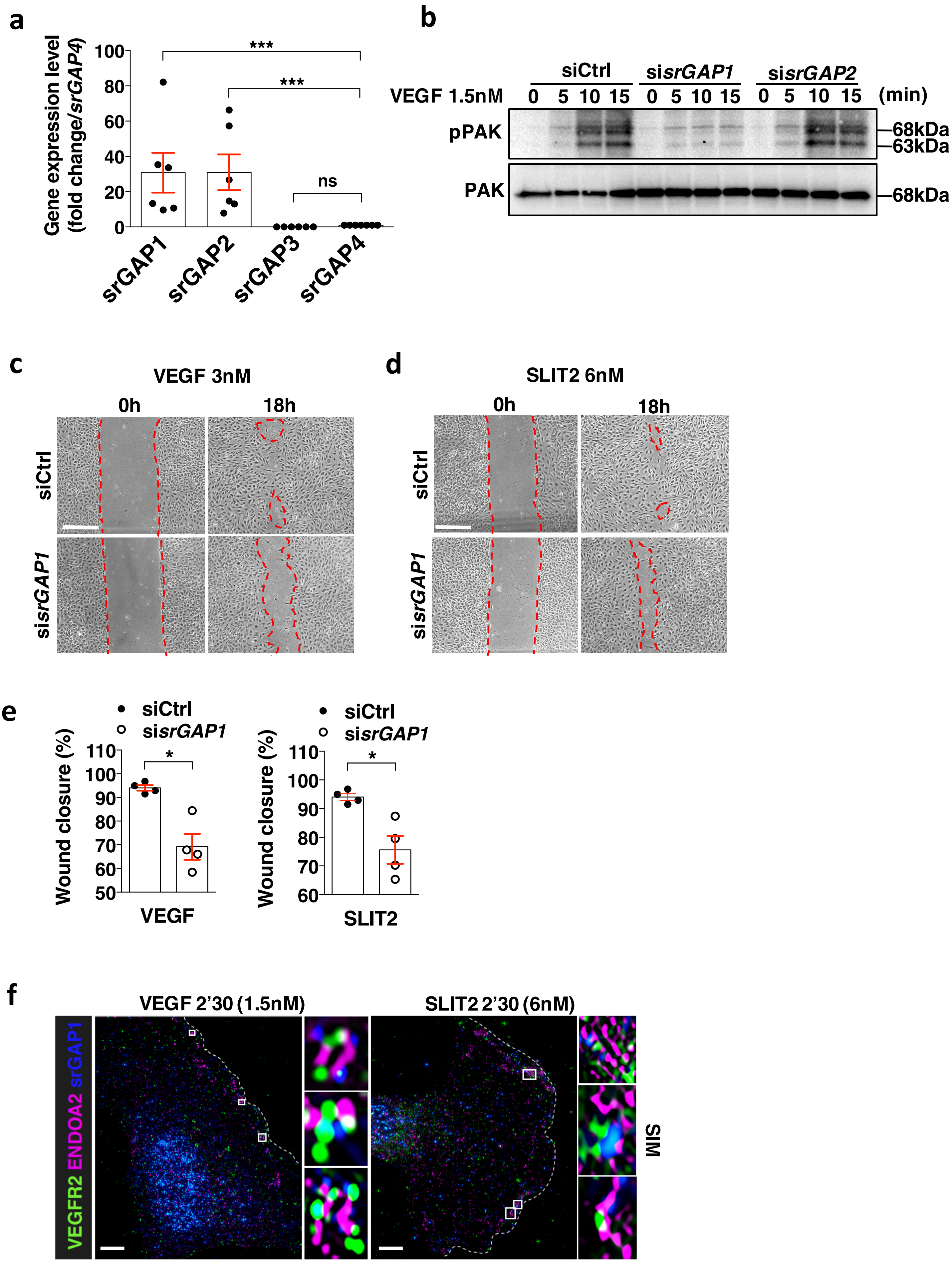

Supplementary Fig.11. srGAP1 function in EC migration.

a, Gene expression of *srGAP 1, 2, 3* and 4 in HUVECs compared to *srGAP4* expression analyzed by qPCR. *Actin* was used as housekeeping gene. (*N*=6 different experiments, one-way ANOVA: ns P<0.05, ***P<0.001). **b**, Western-blot analysis of VEGF-induced (1.5nM) phospho-PAK in HUVECs transfected with the indicated siRNAs. Total PAK expression is shown as loading control. **c and d**, Scratch wound migration in Ctrl and *srGAP1* siRNA silenced HUVECs in response to VEGF (3nM) and SLIT2 (6nM). **e**, Quantification of wound closure (*N*=4 independent experiments; Mann-Whitney U test: *P<0.05). **g,** SIM images of HUVECs stained for VEGFR2, ENDOA2 and srGAP1 after 2'30 VEGF (1.5nM) or SLIT2 (3nM) stimulation. Boxed areas are magnified to highlight VEGFR2/ENDOA2/srGAP1. Error bars represent mean±s.e.m. Scale bars: c and d: 200μm, e: 15μm, f: 20μm

Supplementary Figure 12

a

Fig. 2a

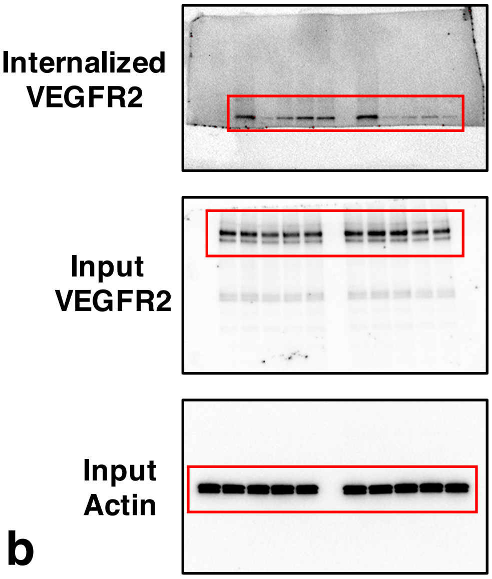

b

Fig. 3i

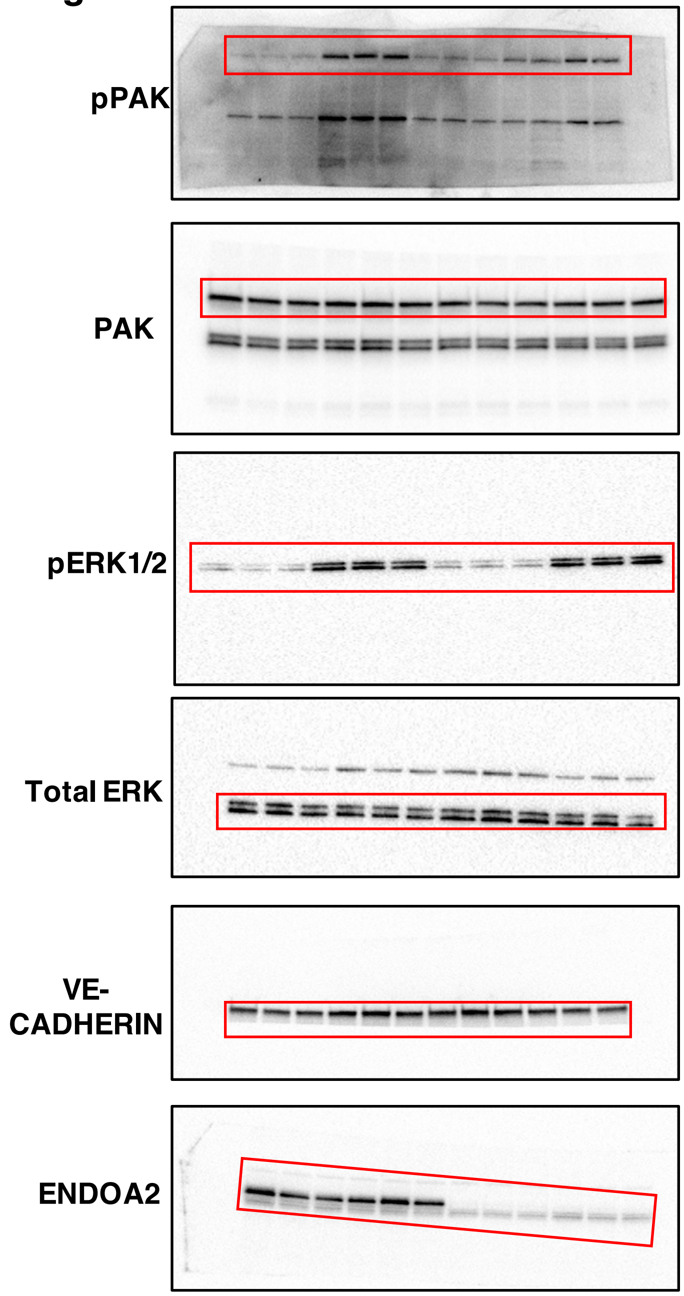

c

Fig. 3g

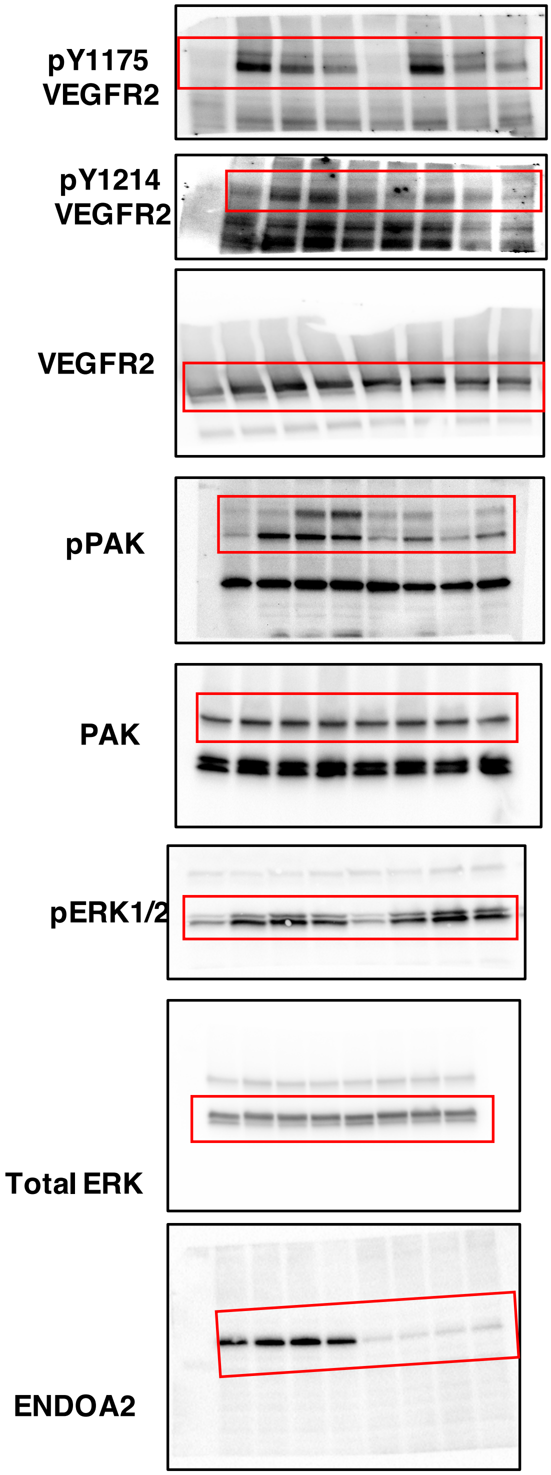

Supplementary Fig.12.
Uncropped western blots from **a**, Fig. 2a ; **b**, Fig. 3i ; **c**, Fig. 3g.

Supplementary Figure 13

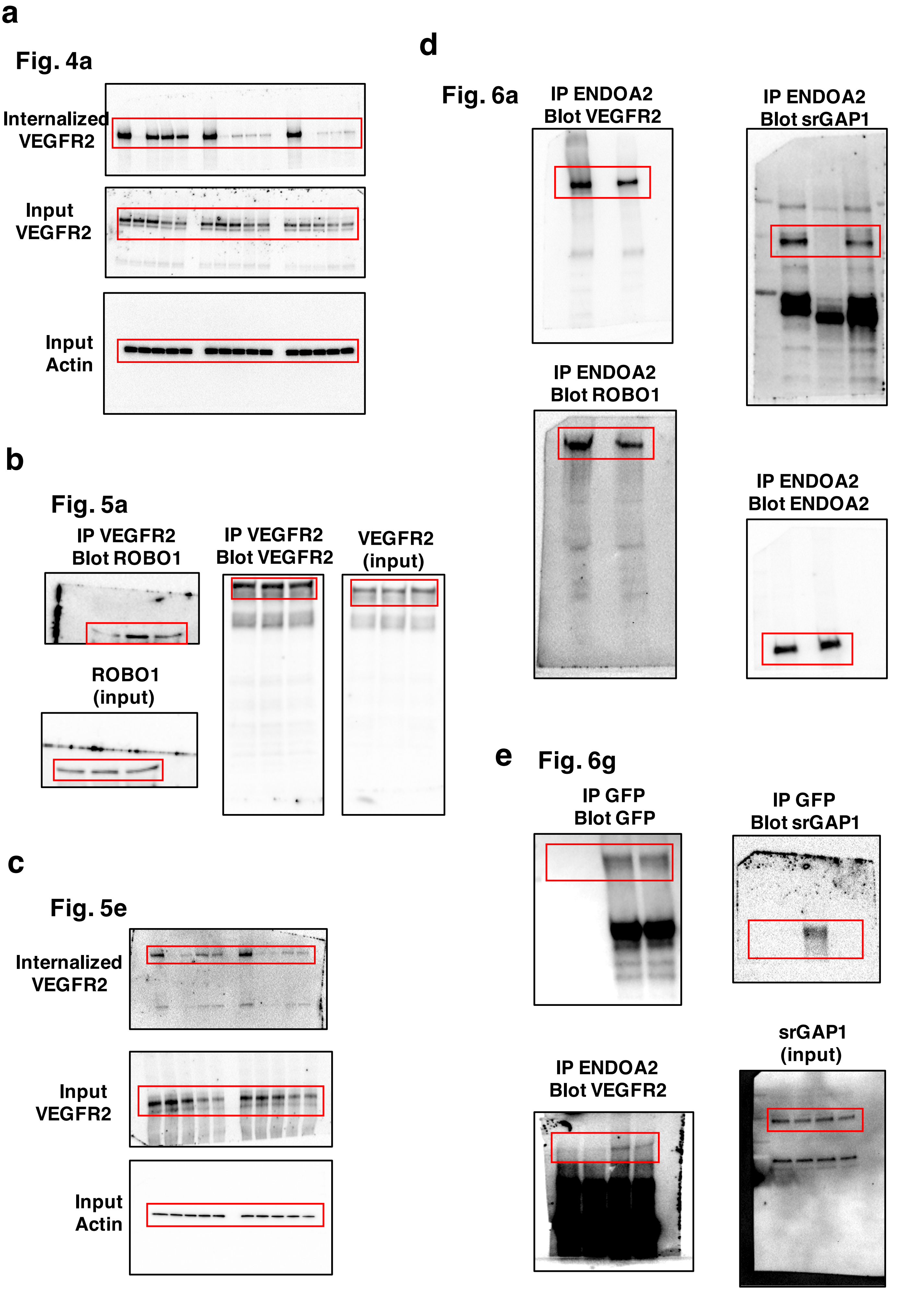

Supplementary Fig.13.
Uncropped western blots from **a**, Fig. 4a; **b**, Fig.5a; **c**, Fig. 5e; **d**, Fig. 6a; **e**, Fig. 6g.
