# Supplementary material for: Endophilin-A2 dependent VEGFR2 endocytosis promotes sprouting angiogenesis
Source: Nat Commun. 2019 May 28;10:2350. doi: 10.1038/s41467-019-10359-x (PMC6538628; doi:10.1038/s41467-019-10359-x)
Supplement: Supplementary file 2 — Reporting Summary [file 41467_2019_10359_MOESM2_ESM.pdf]

nature research

Corresponding author(s): Anne Eichmann

Last updated by author(s): Apr 30, 2019

Reporting Summary

Nature Research wishes to improve the reproducibility of the work that we publish. This form provides structure for consistency and transparency in reporting. For further information on Nature Research policies, see Authors & Referees and the Editorial Policy Checklist.

Statistics

For all statistical analyses, confirm that the following items are present in the figure legend, table legend, main text, or Methods section.

n/a	Confirmed	
☐	☒	The exact sample size (*n*) for each experimental group/condition, given as a discrete number and unit of measurement
☐	☒	A statement on whether measurements were taken from distinct samples or whether the same sample was measured repeatedly
☐	☒	The statistical test(s) used AND whether they are one- or two-sided<br>*Only common tests should be described solely by name; describe more complex techniques in the Methods section.*
☒	☐	A description of all covariates tested
☒	☐	A description of any assumptions or corrections, such as tests of normality and adjustment for multiple comparisons
☐	☒	A full description of the statistical parameters including central tendency (e.g. means) or other basic estimates (e.g. regression coefficient) AND variation (e.g. standard deviation) or associated estimates of uncertainty (e.g. confidence intervals)
☒	☐	For null hypothesis testing, the test statistic (e.g. *F*, *t*, *r*) with confidence intervals, effect sizes, degrees of freedom and *P* value noted<br>*Give P values as exact values whenever suitable.*
☒	☐	For Bayesian analysis, information on the choice of priors and Markov chain Monte Carlo settings
☒	☐	For hierarchical and complex designs, identification of the appropriate level for tests and full reporting of outcomes
☐	☒	Estimates of effect sizes (e.g. Cohen's *d*, Pearson's *r*), indicating how they were calculated

Our web collection on statistics for biologists contains articles on many of the points above.

Software and code

Policy information about availability of computer code

Data collection	No software was used
Data analysis	Image Analysis: Image J 1.51m9<br>Statistical analysis: Prism 7.0

For manuscripts utilizing custom algorithms or software that are central to the research but not yet described in published literature, software must be made available to editors/reviewers. We strongly encourage code deposition in a community repository (e.g. GitHub). See the Nature Research guidelines for submitting code & software for further information.

Data

Policy information about availability of data

All manuscripts must include a data availability statement. This statement should provide the following information, where applicable:

- Accession codes, unique identifiers, or web links for publicly available datasets
- A list of figures that have associated raw data
- A description of any restrictions on data availability

No datasets were generated or analyzed during the current study. The data that support the findings of the study are available on request from the corresponding author A.E.

Field-specific reporting

Please select the one below that is the best fit for your research. If you are not sure, read the appropriate sections before making your selection.

☒ Life sciences ☐ Behavioural & social sciences ☐ Ecological, evolutionary & environmental sciences

For a reference copy of the document with all sections, see nature.com/documents/nr-reporting-summary-flat.pdf

Life sciences study design

All studies must disclose on these points even when the disclosure is negative.

Sample size	For retinal vascular development experiments: 10 to 23 different retinas per group were used.<br>For retinal tip cell polarization experiment: 6 different retinas per group were used.<br>For Oxygen induced retinopathy experiment: 6 retinas per group were used.<br>For Western Blot Analysis (cell surface biotinylation and signaling): 4 to 7 independent experiments were analyzed<br>For cell behavior experiment (proliferation, survival, migration): 3 to 5 independent experiments were analyzed<br>For cell imaging quantification: 8 to 17 different cells from 3 independent experiments were analyzed
Data exclusions	No data exclusions
Replication	all attempts at replication were successful
Randomization	No randomization
Blinding	For in vivo studies blinding was not possible because we had to genotype each animal.<br>For western blot analysis ans cell behavior experiments blinding was not possible because we had to do siRNA transfection.<br>Cell imaging quantification have been done blindly.

Reporting for specific materials, systems and methods

We require information from authors about some types of materials, experimental systems and methods used in many studies. Here, indicate whether each material, system or method listed is relevant to your study. If you are not sure if a list item applies to your research, read the appropriate section before selecting a response.

Materials & experimental systems

n/a	Involved in the study	
☐	☒	Antibodies
☐	☒	Eukaryotic cell lines
☒	☐	Palaeontology
☐	☒	Animals and other organisms
☒	☐	Human research participants
☒	☐	Clinical data

Methods

n/a	Involved in the study	
☒	☐	ChIP-seq
☒	☐	Flow cytometry
☒	☐	MRI-based neuroimaging

Antibodies

Antibodies used	EndophilinA2 (sc-365704, Santa Cruz), CLATHRIN (4796P, Cell Signaling), anti-Robo1 (MAB7118, R&D systems), anti-GM130 (610823, BD), anti-NG2 (AB5320, Millipore), anti-Desmin (AT3844, NovusBio), anti-VECadherin (Santa Cruz, Sc6458), (anti-Collagen IV (AB769, Millipore), anti-ERG1/2/3 (SC353, Santa Cruz), anti-α-smooth muscle actin CY3 (CY3-SMA, C6198, Sigma), anti cleaved caspase-3 (9661S, Cell Signaling), anti-Podocalyxin (AF1556, R&D systems), Dapi (D1306, Life Technologies), anti-pVEGFR2 1175 (2478, Cell Signaling), anti-pVEGFR2 1214 (2477, Cell Signaling), anti-p44/42 MAP kinase (phospho-ERK, 9106, Cell Signaling), anti-p44/42 MAP kinase (total ERK, 9102, Cell Signaling), anti-pPAK1 (Thr423)/PAK2 (Thr402) (2601S, Cell Signaling), anti-PAK1/2/3 (2604, Cell Signaling), anti-srGAP1 (ab76926, Abcam), anti-p38 MAPK (8690T, Cell Signaling), anti-phosp38 MAPK (4511T, Cell Signaling), anti-pMLC2 (3671S, Cell Signaling), anti-VEGFR2 (9698, Cell signaling), anti-actin (A1978, Sigma), anti-Calretinin (MAB1568, Millipore), anti-endomucin (HM1108, Hycult), anti-GFAP (ZO334, Dako). Appropriate secondary antibodies were conjugated to horseradish peroxidase (Vector Laboratories) or fluorescently labeled (Life Technologies). IsolectinB4 (I21411), Dapi (D1306) and phalloidin568 were purchased from Life Technologies.
Validation	*Describe the validation of each primary antibody for the species and application, noting any validation statements on the manufacturer's website, relevant citations, antibody profiles in online databases, or data provided in the manuscript.*

Eukaryotic cell lines

Policy information about cell lines

Cell line source(s)	HUVECs were obtained from the Yale University Vascular Biology and Therapeutics Core Facility
Authentication	The name of the cell line used was identified
Mycoplasma contamination	HUVECs were not tested for mycoplasma contamination
Commonly misidentified lines (See ICLAC register)	*Name any commonly misidentified cell lines used in the study and provide a rationale for their use.*

Animals and other organisms

Policy information about studies involving animals; ARRIVE guidelines recommended for reporting animal research

Laboratory animals	- EndoA2-/- mice: strain S129, male and female were used at P5 (retinal vasculature development studies) and at P17 (OIR).<br>- Robo1-/-Robo2fl/flCDH5CRE ERT2 mice , strain C57BL6, male and female were used at P5
Wild animals	*Provide details on animals observed in or captured in the field; report species, sex and age where possible. Describe how animals were caught and transported and what happened to captive animals after the study (if killed, explain why and describe method; if released, say where and when) OR state that the study did not involve wild animals.*
Field-collected samples	*For laboratory work with field-collected samples, describe all relevant parameters such as housing, maintenance, temperature, photoperiod and end-of-experiment protocol OR state that the study did not involve samples collected from the field.*
Ethics oversight	All animal procedures were reviewed and approved by the Institutional Animal Care Use Committee of Yale University

Note that full information on the approval of the study protocol must also be provided in the manuscript.
